# Health interventions and stakeholder roles in neonatal mortality reduction within low-income countries: A scoping review

**DOI:** 10.1016/j.puhip.2026.100847

**Published:** 2026-08-21

**Authors:** Mia Rose, Anika Kreutzberg, Miriam Pospisil, Vincent Findeiss

**Affiliations:** a Berlin School of Public Health, Charité - Universitätsmedizin Berlin, Berlin, Germany; b Department of Health Care Management, Berlin University of Technology, Berlin, Germany

**Keywords:** Interventions, Newborn care, Neonatal mortality, Multi-stakeholder partnerships, Low-income countries, Sustainable development goals

## Abstract

**Objectives:**

Neonatal mortality continues to represent a major global health challenge, with the highest mortality rates being observed in low-income countries (LICs). Despite the adoption of Sustainable Development Goal (SDG) 3.2, there has been limited progress in reducing deaths due to preterm birth complications, birth asphyxia, and infections among newborns. In order to inform future policy and implementation strategies, it is essential to understand which neonatal health interventions have been implemented since 2015 and which stakeholders have been involved in their implementation and funding. This scoping review maps health interventions implemented in low-income countries (LICs) since the adoption of Sustainable Development Goal (SDG) 3.2 in 2015, targeting key causes of neonatal mortality: prematurity, birth asphyxia, and infections. It also explores multi-stakeholder partnerships supporting implementation and financing.

**Study design:**

Scoping review.

**Methods:**

Medline Ovid, Embase Ovid, CINAHL, and the Cochrane Library were searched for peer-reviewed literature, and Google Scholar was searched for grey literature, covering an intervention period from 2015 to 2025. The review followed the Joanna Briggs Institute methodology and was reported according to PRISMA-ScR guidelines. A protocol was prospectively registered on the Open Science Framework (OSF-ID cdpr2).

**Results:**

22 studies met the inclusion criteria. Interventions were clustered into six thematic categories: quality of neonatal care improvement initiatives (n = 6), neonatal resuscitation training (n = 5), Kangaroo Mother Care implementation strategies (n = 4), pharmaceutical and nutritional interventions (n = 4), mHealth interventions (n = 1), and thermal and respiratory support (n = 2). Implementation was largely facilitated through multi-stakeholder partnerships involving public health facilities, academic institutions, non-profit organizations (NPOs), non-governmental organizations (NGOs), and governmental authorities.

**Conclusions:**

Multi-stakeholder partnerships play a crucial role in the implementation of neonatal health interventions in LICs, with public and non-profit actors most frequently engaged. However, limited reporting on stakeholder roles and financing mechanisms raises concerns about long-term sustainability. Greater transparency in implementation and funding processes is needed to accelerate progress toward achieving SDG 3.2 by 2030.

**What this study adds**

• This scoping review provides the first comprehensive mapping of maternal and neonatal health interventions implemented in low-income countries (LICs) since the adoption of SDG 3.2, with a specific focus on the three leading causes of neonatal mortality.
• The findings show a dominance of facility-based, quality improvement–oriented interventions and limited diversity in intervention approaches across settings.
• This scoping review highlights substantial gaps in reporting on stakeholder roles and financing mechanisms, alongside a strong reliance on externally driven partnerships.

**Implications for policy and practice**

• The findings of this scoping review highlight the need to enhance local ownership and sustainability by increasing the involvement of domestic stakeholders in leadership, funding, and implementation.
• The results suggest the importance of increasing investment in context-specific, community- and system-level interventions to complement the current focus on facility-based approaches and improve equitable access.
• The study findings indicate the need to strengthen transparency and standardised reporting of funding streams, clearly distinguishing between research funding, implementation financing, and in-kind support.

## 1 Introduction

Improving newborn health remains a major public health challenge in low- and middle-income countries (LMICs) [1]. In 2022, 47 % of global under-five deaths occurred within the first 28 days of life [2].

Despite a 44 % global reduction in neonatal mortality since 2000, rates remain high in low-income countries (LICs) [2]. In 2022, the UN Inter-agency Group for Child Mortality Estimation (UN IGME) reported a neonatal mortality rate of 24.9 per 1000 in LICs, compared to 2.7 per 1000 in Europe and North America [3]. Neonatal mortality remains particularly high in sub-Saharan Africa, recording the highest global neonatal mortality rate at 27 deaths per 1000 live births in 2022 [2,4]. Prematurity, birth asphyxia, and neonatal infections account for most preventable neonatal deaths [[5], [6], [7]]. Health interventions such as antenatal corticosteroids to prevent neonatal respiratory distress syndrome in preterm infants, umbilical cord cleansing with chlorhexidine to prevent infections, and Kangaroo Mother Care for preterm infants have demonstrated great potential to reduce mortality [8,9].

In 2015, the UN 2030 Agenda for Sustainable Development set a target to reduce preventable neonatal mortality to 12 deaths per 1000 live births by 2030 (Sustainable Development Goal (SDG) 3.2) [10]. However, 69 of the 191 UN member states that signed the agenda are expected to miss this target [11] due to persistent gaps in workforce capacity [12] and sustainable financing and social protection [13,14]. The agenda highlights the role of multi-stakeholder partnerships (SDG 17.16) in mobilising knowledge, technology, and resources [10]. Complex neonatal health interventions often require collaborative approaches, involving health-care providers, researchers, ministries of health, non-profit organizations and local authorities [15].

Previous systematic reviews have identified effective interventions to reduce neonatal mortality in LMICs, including preterm birth prevention [16], neonatal resuscitation training [17], and community-based antibiotic treatment for severe neonatal infections [18]. Other reviews focused on humanitarian and conflict settings [19,20] or on interventions implemented prior to the adoption of SDG 3.2 [21]. Moon et al. (2024) mapped stakeholders involved in addressing preterm birth, identifying 38 national and global organizations, mostly knowledge-sharing, advocacy, and implementing bodies, largely based in high-income countries [15]. To our knowledge, no synthesis maps interventions targeting prematurity, birth asphyxia, or infections in LICs since SDG 3.2 while also outlining the multi-stakeholder partnerships supporting implementation and financing. This scoping review has two aims: (1) it maps health interventions implemented in LICs since 2015 that address prematurity, birth asphyxia, and infections [[5], [6], [7]] and (2) it describes the multi-stakeholder partnerships that supported implementation and financing.

## 2 Methods

A scoping review was conducted given the broad and exploratory nature of the research questions and the heterogeneity of the available evidence in terms of study design, intervention types, and reporting of stakeholder involvement. This approach enabled the mapping of diverse interventions and multi-stakeholder partnerships across low-income country contexts. The review follows the methodological guidance of the Joanna Briggs Institute (JBI) [22] and reporting conforms to PRISMA extension for scoping reviews (PRISMA-ScR) guidelines [23]. The study protocol was prospectively registered and is publicly available on the Open Science Framework (OSF-ID cdpr2), but was not published in a peer-reviewed journal. Any deviations from the protocol are reported. The development of the research questions, the search strategy, and eligibility criteria were based on the following PCCT framework (population, concept, context, time).

• Population: newborns (≤28 days of age) [24].
• Concept: health interventions to reduce neonatal mortality
• Context: LICs [25].
• Time**:** interventions implemented in 2015 or later

The PCCT framework was used to incorporate a temporal restriction (interventions implemented from 2015 onwards), thereby ensuring a focus on recent evidence aligned with the Sustainable Development Goals and current neonatal health strategies.

### 2.1 Eligibility criteria

Eligible sources were primary study designs reporting neonatal mortality as an outcome and addressing at least one of the three leading causes of neonatal mortality: prematurity, birth asphyxia, or infections [[5], [6], [7]]. Excluded were reviews, study protocols, and conference abstracts.

Interventions had to run for at least 6 months or report outcomes ≥ 6 months after baseline and had to be initiated between 2015 and 2025. These eligibility criteria were applied to ensure that included interventions had sufficient time for implementation and for measurable effects on neonatal outcomes to emerge. Accordingly, shorter intervention durations may be less likely to capture meaningful and sustained effects on neonatal outcomes.

Studies had to be conducted in low-income countries (LICs) (World Bank FY2025 classification) [25], and published in English. Multicentre studies were eligible if at least one study site was located in a LIC. Where possible, LIC-specific data were extracted for analysis. Studies for which full text could not be accessed were excluded.

### 2.2 Search strategy

We searched Medline Ovid, Embase Ovid, CINAHL, and the Cochrane Library for peer-reviewed literature and used Google Scholar for grey literature on 20 February 2025. The search strategy is consistent with JBI guidelines and is provided in supplement A2 and A3. Search strings included keywords such as “infant, newborn”, “health intervention”, and “neonatal mortality”, combined with LIC country names. In Google Scholar, separate keyword combinations were searched per LIC, screening the first 10 results per combination. A consistent date filter (2015–2025) was applied across all databases to restrict the search period, capturing the timeframe following the adoption of the Sustainable Development Goals.

### 2.3 Evidence screening and selection

Two reviewers (MR and MP) independently screened titles and abstracts. Discrepancies were resolved through discussion until consensus was reached on whether to include the study for full-text review. One reviewer (MR) screened all full texts against eligibility criteria. A random sample of full texts (n = 15; 20 %) was independently screened by both reviewers (MR and MP) to assess consistency. An agreement rate of 100 % (n = 15) was identified.

### 2.4 Data extraction, charting, and synthesis

Data extraction was organized into three thematic sections, with interventions recorded first by thematic category, then by the stakeholders involved in their implementation, and finally by source of funding.

Thematic categories were derived inductively but informed by established intervention frameworks. In particular, interventions were interpreted in relation to a catalogue of 61 reproductive, maternal, newborn, and child health (RMNCH) interventions classified as essential by the World Health Organization [26]. This enabled the grouping of similar intervention approaches while still allowing new themes to emerge from the data.

Stakeholders were coded using a three-step approach adapted from World Health Organization guidance [27]. First, stakeholders were identified and listed by name. Second, the level of involvement for each stakeholder was recorded. Third, the specific “stake” or role and sector of each stakeholder was described.

Consistent with scoping review guidelines, no critical appraisal of study quality or risk of bias assessment was undertaken, as the objective was to map existing evidence rather than evaluate study outcomes. Consistent with scoping review guidelines [22], no critical appraisal of study quality or risk of bias assessment was undertaken. However, studies were classified by study design to provide a basic indication of their position within the evidence hierarchy.

## 3 Results

### 3.1 Included studies

A flow chart illustrating the selection and screening process of studies is presented in Fig. 1. Of 3926 studies 2321 remained after deduplication for title and abstract screening. 76 studies were selected for full-text screening. 54 studies were excluded based on the predefined eligibility criteria. For each study the specific reasons for exclusion are provided in supplement A4.

**Fig. 1:**
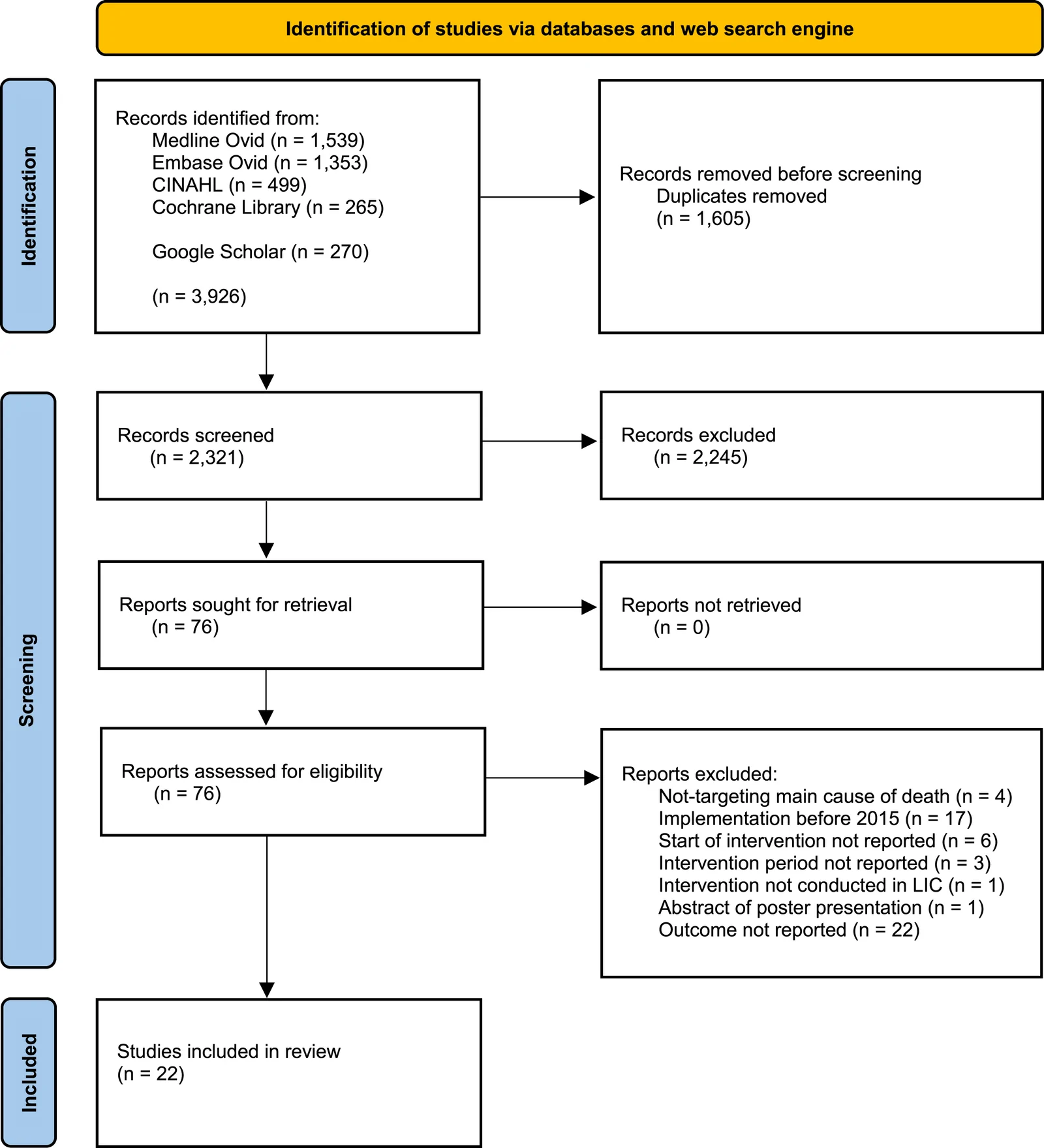
Flow chart for study selection, adapted from PRISMA 2020 statement [28].

Ultimately, 22 studies were included in the final data extraction and analysis. The extracted data from all included studies are presented in Table 1.

**Table 1:** Summary of included studies.

ID	Study	Country	Study period	Study design	Target cause	Description of intervention	Intervention setting	Intervention level of involvement	Intervention partnership actors	Intervention funding reported
**1**	Arya et al., 2021 [29]	Malawi Ghana, India, Nigeria, Tanzania	November 2017 - January 2020	Multi-country RCT	Prematurity/low birth weight	Immediate initiation of KMC for neonates with a birthweight between 1000 and 1200 g shortly after birth and before clinical stabilization, compared to conventional care with delayed KMC	5 public tertiary-level hospitals	Facility-level intervention	Academic research institutions WHO	no
**2**	Baumgartner et al., 2021 [30]	Uganda	May 2017 - August 2018	Quasi-experimental pre-post observational study	Infections, birth asphyxia	Clinical and management quality improvement programme by NGO LifeNet International using monthly on-site training, team-based behaviour change, and clinical mentoring on infection prevention, neonatal resuscitation, and postpartum haemorrhage	6 rural, faith based, not-for-profit primary healthcare facilities in the greater Maka area	Facility-level intervention	Non-profit NGOs Public health facilities & hospitals	yes
**3**	Bountogo et al., 2023 [31]	Burkina Faso	April 2019 - December 2020	RCT	Neonatal infections/low birth weight	Single-dose oral azithromycin (20 mg/kg) administered to low birthweight or underweight neonates aged 8–27 days, compared to placebo	44 public primary healthcare facilities in 5 regions of Burkina Faso	Facility-level intervention	Academic research institutions	no
**4**	Brotherton et al., 2021 [32]	The Gambia	May 2018 - April 2020	RCT	Prematurity/low birth weight	Early initiation of continuous KMC within 24 h of admission for mildly to moderately unstable neonates <2000g, compared to standard care	Public tertiary-level referral Edward Fracis Small Teaching Hospital	Facility-level intervention	Academic research institutions Public health facilities & hospitals	no
**5**	Draiko et al., 2019 [33]	South Sudan	February 2017 - August 2018	Pre-post intervention study	Birth asphyxia	Health workers received *Helping Babies Breathe* training, a curriculum to improve neonatal resuscitation skills in low-resource setting	2 public tertiary-level referral teaching hospitals in Juba and Wau	Facility level intervention	Academic research institutions Public health facilities & hospitals	no
**6**	Gidey et al., 2023 [34]	Ethiopia	June 2017 - February 2018	Prospective cohort study	Prematurity/low birth weight	Post-discharge practice of KMC among mothers of low birth weight infants (<2000g)	Public tertiary-level hospital (Ayder) and public secondary-level general hospital (Mekelle) in the city of Mekelle	Community-level intervention	Public health facilities & hospitals WHO	yes
**7**	Hendrixson et al., 2021 [35]	Sierre Leone	February 2017 - February 2020	RCT	Prematurity, Infection/low birth weight	Package of nutritional and anti-inflammatory interventions for undernourished pregnant women to control infection and promote newborn growth (weight and length)	43 public antenatal clinics in Western Rural Area and Pujehun districts	Facility-level intervention	Academic research institutions Local governmental authorities Ministries of health Non-profit NGOs Public Health Facilities & Hospitals	no
**8**	Innerdal et al., 2020 [36]	Mali	January 2015 -December 2018	Pre-post intervention study	Birth asphyxia	Implementation of the *Helping Babies Breathe* training for birth attendants	District hospital in Kati district in Mali	District-level intervention	Academic Research Institutions Local governmental authorities Non-profit NGOs Public health facilities & hospitals	no
**9**	Kavi et al., 2023 [37]	DRC Guatemala, India, Kenya, Pakistan, Zambia	March 2016 - April 2019	Secondary analysis of an RCT	Prematurity/hypertensive disorders of pregnancy	Daily low-dose aspirin (81 mg) administered to nulliparous women to prevent preterm birth related to hypertensive disorders	Primary healthcare centres and hospital-based clinics	Facility-level intervention	Academic research institutions	no
**10**	Mwalweni et al., 2024 [38]	Malawi	June 2023 - February 2024	Retrospective pre-post intervention study	Birth asphyxia	Implementation of a criterion-based audit to evaluate and improve quality of care for neonatal birth asphyxia	Public primary care Balaka district Hospital	District-level intervention	Local governmental authorities Public health facilities & hospitals	no
**11**	Nishimwe et al., 2022 [39]	Rwanda	February 2019 - March 2020	Pre-post intervention study	Birth asphyxia	Implementation of the *Safe Delivery* mHealth Application to support clinical decision-making in maternal and neonatal care by providing nurses and midwives with basic emergency obstetric and newborn care guidelines	2 public primary care district hospitals in Rwanda (Masaka hospital and Nyamata hospital)	District-level intervention	Academic research institutions Ministries of health Private non-profit foundations Public health facilities & hospitals	no
**12**	Okello et al., 2019 [40]	Uganda	May 2015 - December 2017	Retrospective pre-post intervention study	Prematurity/low birth weight	Introduction of low-cost bubble CPAP therapy for very low birth weight neonates (<1500 g)	Public secondary-level referral Mbale Regional Referral Hospital	District-level intervention	Academic research institutions Public health facilities & hospitals	yes
**13**	Ouedraogo et al., 2022 [41]	Burkina Faso	January 2015 - December 2018	Retrospective pre-post intervention study	Prematurity, birth Asphyxia	*Neonatal Essential Survival Technology* intervention programme including neonatal resuscitation training, improved clinical practices, and equipment provision to improve quality of neonatal care	Tertiary-level private Saint Camille Hospital Ouagadougou	Facility-level intervention	Local governmental authorities Private non-profit foundations Public health facilities & hospitals	yes
**14**	Patterson et al., 2022 [42]	DRC	January 2017 - June 2020	Pre-post intervention study (retrospective control group, prospective intervention group)	Birth asphyxia/stillbirth misclassification	*Helping Babies Breathe* neonatal resuscitation training combined with continuous electronic heart rate monitoring using dry-electrode C-shaped NeoBeat™ device for non-breathing newborns	3 urban health facilities (one public, two catholic) in Kinshasa	Facility-level intervention	Academic research institutions Non-profit NGOs Public health facilities & hospitals	no
**15**	Roca et al., 2023 [43]	The Gambia, Burkina Faso	October 2017 - May 2021	RCT	Neonatal infection/sepsis	Single-dose intrapartum oral azithromycin (2g) administered to women during labour, compared to placebo	2 public health facilities close to Banjul, The Gambia, and 8 health facilities in the rural districts of Nanoro and Yako, Burkina Faso	Facility-level intervention	Academic research institutions Public health facilities & hospitals	no
**16**	Tadesse et al., 2021 [44]	Ethiopia	Jun 2016 - March 2019	Pre-post mixed methods intervention study	Birth asphyxia	Implementation of a low-dose/high-frequency training programme to establish a nurse- and midwife-led neonatal resuscitation service, based on evidence-based curricula	Public tertiary-level referral St. Paul's Hospital Millennium Medical College	Facility-level intervention	Academic research institutions Ministries of health Non-profit NGOs	yes
**17**	Tumukunde et al., 2024 [45]	Uganda	October 2019 - July 2022	RCT and economic evaluation	Prematurity/low birth weight	Immediate KMC initiated before clinical stabilization in neonates <2000g, compared to standard care	5 public hospitals in Uganda	Facility-level intervention	Academic research institutions Public health facilities & hospitals	no
**18**	Tuyisenge et al., 2021 [46]	Rwanda	April 2017 - September 2019	Pre-post quasi-experimental study	Prematurity, birth asphyxia	Multi-component quality improvement strategy including clinical mentoring, staff training, provision of equipment and supplies, and peer-to-peer learning through collaborative learning sessions (*All Babies Count bundle)* to improve neonatal care	7 public rural district hospitals	District-level intervention	Local governmental authorities Ministries of health Non-profit NGOs Public health facilities & hospitals	yes
**19**	Umunyana et al., 2020 [47]	Rwanda	January 2016 - June 2018	Pre-post mixed methods study	Birth asphyxia	Implementation of a system-oriented practice improvement package, including *Helping Babies Breathe* training, clinical mentorship, and quality improvement activities	12 public hospitals and 160 public health centres in 10 districts	District-level intervention	Academic research institutions Ministries of health Public health facilities & hospitals	no
**20**	Uwamariya et al., 2021 [48]	Rwanda	November 2019 - July 2020	Cluster-randomized stepped-wedge trial	Prematurity/hypothermia	Development and rollout of the *Dream Warmer*, a reusable, non-electric, low-cost infant warming device	10 district hospitals	District-level intervention	Academic research institutions Ministries of health Non-profit NGOs Public health facilities & hospitals	no
**21**	Walker et al., 2020 [49]	Uganda Kenya	June 2015 - April 2019	Cluster-RCT	Prematurity/low birth weight	Quality-of-care improvement package targeting preterm birth by reinforcing existing data systems to enhance data use, strengthening provider skills and team communication, and catalysing system-level improvements	4 public facilities in in the Busoga region in eastern Uganda and 16 public facilities in Migori County in western Kenya	District-level intervention	Academic research institutions Community health volunteers Local governmental authorities Ministries of health	no
**22**	Wotango et al., 2025 [50]	Ethiopia	March 2022 -January 2024	Quality improvement project with PDSA cycles based on Model for Improvement framework	Prematurity	Quality improvement intervention including implementation of a standardised CPAP transport protocol and infection prevention checklist, transport of preterm neonates from the labour ward to neonatal intensive care unit with CPAP, and use of a CPAP device with integrated oxygen concentrator, blender, and compressor.	Public Gandhi Memorial Hospital, Addis Ababa	Facility-level intervention	Academic research institutions Local governmental authorities Public health facilities & hospitals	no

Abbreviations: CPAP = continuous positive airway pressure, DRC = Democratic Republic of Congo, ID = Identifier, KMC = Kangaroo Mother Care, NGO = Non-Governmental Organization, PDSA = Plan, Do, Study, Act, RCT = randomized controlled trial.

### 3.2 Identified interventions for reducing neonatal mortality

Each of the 22 studies evaluated distinct interventions. Study designs included randomized controlled trials (n = 9; 40.9 %) and quasi-experimental pre-post studies (n = 11; 50 %). Most interventions started in 2017 (n = 8; 36.4 %). Fig. 2 visualizes the geographical distribution of interventions. Among the 25 low-income countries defined by the World Bank FY2025 classification, only four (Afghanistan, Yemen, the Syrian Arab Republic, and the Democratic People's Republic of Korea) are located outside sub-Saharan Africa. Across the included studies, no interventions were identified from these settings, resulting in an exclusive geographical concentration of evidence in sub-Saharan Africa, with the highest number of studies conducted in Rwanda and Uganda (each n = 4; 18.2 %).

**Fig. 2:**
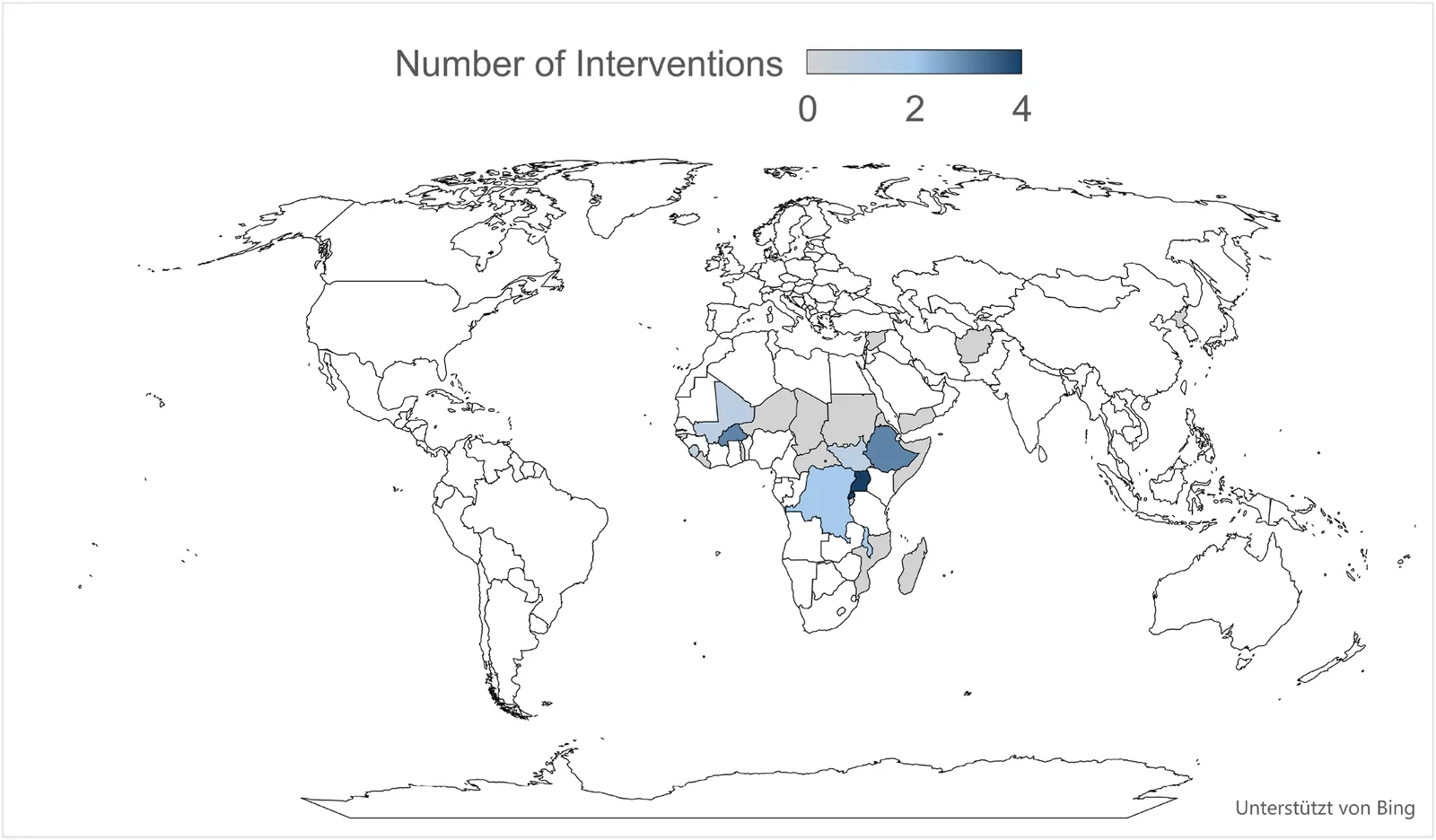
Geographical distribution of interventions included from low-income countries. Note: all LICs, as classified by the World Bank for the 2025 fiscal year, are highlighted in grey.

Across the 22 included studies, most interventions were implemented at the **facility level (n = 13; 59.1 %)**, primarily through clinical trials, quality improvement initiatives, and health worker training within hospitals or maternity services. A smaller number of studies involved **district-level implementation (n = 8; 6.4 %),** typically through scaled or multi-facility quality improvement packages or cluster-based delivery models, while **community-level interventions were rare (n = 1; 4.6 %).**

Interventions were grouped into six thematic categories, as shown in Table 2. Six interventions (27.3 %) were classified under *quality of neonatal care improvement initiatives* [30,38,41,46,49,50]**.** These interventions comprised process-level improvements, including on-site clinical and management training [30], reinforcement of existing data systems to enhance data utilisation [49], the introduction of technology-supported care packages such as the *Neonatal Essential Survival Technology* programme [41], and multi-component quality improvement (QI) strategies such as the *All Babies Count* programme [46], integrating clinical mentorship, staff training, equipment provision, and peer-to-peer learning. Structure-level improvements included criterion-based audits aimed at enhancing care for neonatal asphyxia [38] and the implementation of standardised continuous positive airway pressure (CPAP) protocols and infection prevention checklist [50].

**Table 2:**
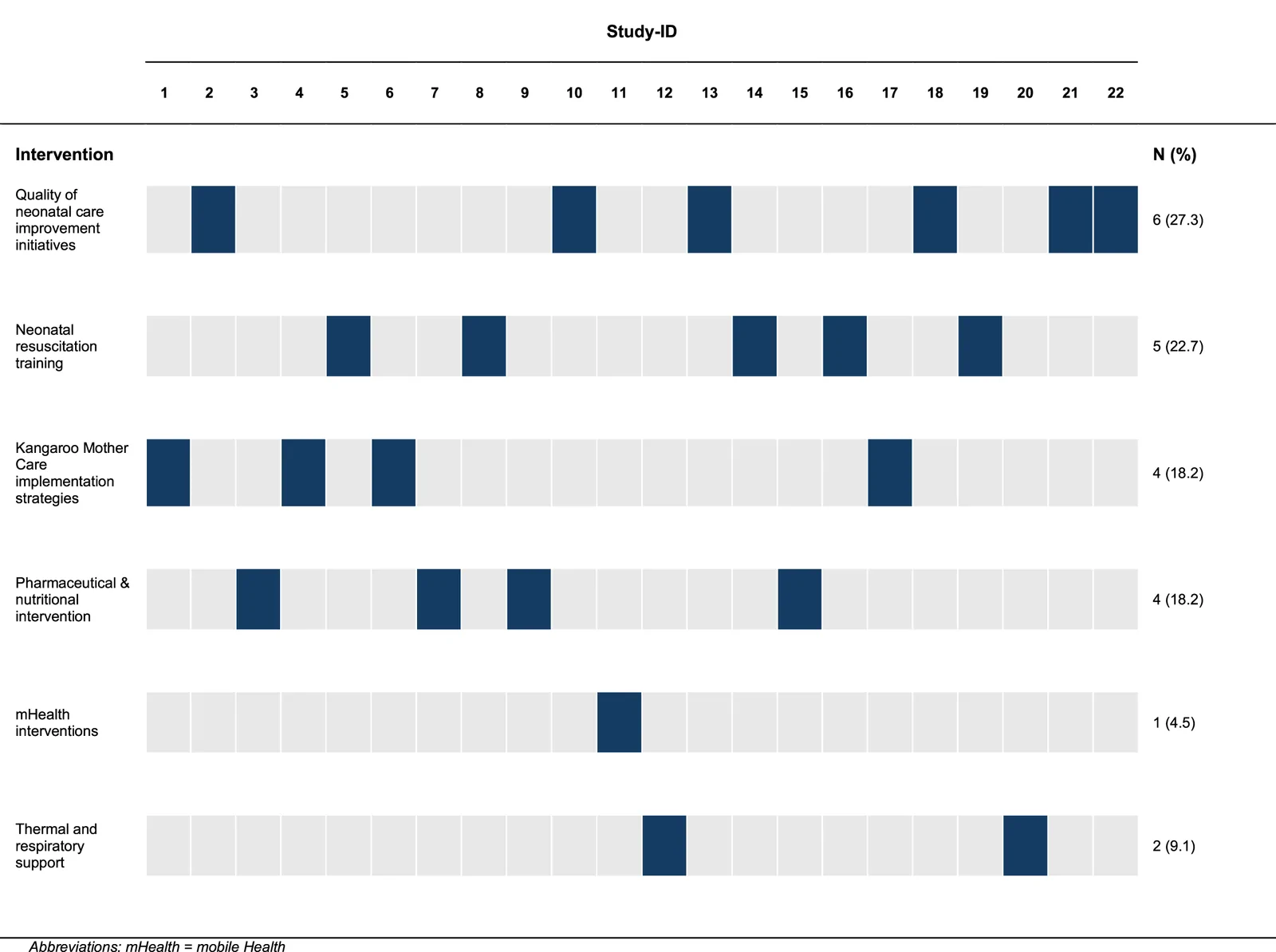
Interventions by thematic category.

Five interventions (22.7 %) were categorised under *neonatal resuscitation training* [33,36,42,44,47]. All focused on building workforce capacity to effectively manage birth asphyxia and improve immediate newborn care. Three studies investigated the *Helping Babies Breathe* programme, with initial and refresher training for birth attendants at hospitals and health facilities [33,36,47]. Some interventions expanded the scope by integrating low-dose/high-frequency training models [44], continuous clinical mentorship [47], or system-oriented quality improvement components [47]. Patterson et al. (2022) introduced dry-electrode heart-rate monitoring (NeoBeatTM) to reduce misclassification of stillbirths in non-breathing newborns [42].

In the category *Kangaroo Mother Care (KMC) implementation strategies*, all interventions (n = 4; 18.2 %) optimized timing and continuity of KMC for low birth weight and preterm neonates [29,32,34,45], assessing early or immediate initiation of KMC [29,32,45] at birth [29], within 24 h of admission [32], and before clinical stabilization [29,45], compared to delayed standard care. Gidey et al. (2023) further included post-discharge continuation of KMC practices at home among mothers who had initiated the care at health facilities [34].

Four interventions (18.2 %) were categorised as *pharmaceutical and nutritional interventions* [31,35,37,43]. They focused on preventing neonatal complications like infections [31,35,43], hypertensive disorders [37], or low birth weight [31,35], through drug- and nutrition-based strategies, including a single-dose oral azithromycin given to neonates or intrapartum to women to prevent infection [31,43], as well as a low-dose aspirin for nulliparous women to reduce the risk of preterm birth associated with hypertensive disorders [37]. Hendrixson et al. (2021) tested a nutritional and anti-inflammatory supplementation package targeting undernourished pregnant women to improve newborn growth [35].

One out of 22 interventions (4.5 %) was categorised as *mHealth intervention* targeting structural improvements in maternal and neonatal care [39]. The intervention encompassed an mHealth decision-support application, the *Safe Delivery Application,* which supports nurses and midwives in clinical decision-making by providing access to evidence-based guidelines for basic emergency obstetric and newborn care [39].

The two interventions (9.1 %) in category *thermal and respiratory* support provided life-saving stabilization in facilities with limited access to advanced neonatal equipment [40,48], addressing the needs of preterm and very low birth weight infants including low-cost bubble CPAP therapy to support respiratory function [40] and the *Dream Warmer*, a reusable, non-electric thermal device designed to prevent neonatal hypothermia [48].

Across countries, intervention types clustered around a limited set of recurring approaches. Facility-based interventions, particularly KMC, neonatal resuscitation training, and quality improvement packages, were most frequently implemented across East African settings such as Rwanda and Uganda [30,33,39,44,46,47,49]. In contrast, studies conducted in West African countries, including Burkina Faso and Sierra Leone, more commonly evaluated biomedical and preventive interventions, such as antibiotic prophylaxis and nutritional supplementation [31,35,41,43]. Multi-country studies predominantly focused on standardized clinical interventions, including KMC and pharmacological strategies [29,37,43,49]. Across all settings, interventions were primarily implemented at the facility level, with fewer studies adopting district- or community-level approaches, indicating a strong focus on strengthening care delivery within health facilities rather than broader system or community-based interventions [30,36,40,49,50].

### 3.3 Stakeholders involved in implementing interventions

Implementation typically involved multi-stakeholder partnerships, as shown in Table 3. Across the included studies, public health facilities and hospitals (n = 17; 77.3 %) [30,[32], [33], [34], [35], [36],[38], [39], [40], [41], [42], [43],[45], [46], [47], [48],^50^] and academic research institutions (n = 17; 77.3 %) [29,[31], [32], [33],[35], [36], [37],^39^,^40^,[42], [43], [44], [45],[47], [48], [49], [50]] were the most frequently involved stakeholders, followed by ministries of health (n = 7; 31.8 %) [35,39,44,[46], [47], [48], [49]], local governmental authorities (n = 7; 31.8 %) [35,36,38,41,46,49,50], and non-profit NGOs (n = 7; 31.8 %) [30,35,36,42,43,46,48]. Additional actors included multilateral organizations such as WHO (n = 2; 9.1 %) [29,34], private non-profit foundations (n = 2; 9.1 %) [39,41], and community health volunteers (n = 1; 4.5 %) [49]. No study reported the involvement of private for-profit providers.

**Table 3:**
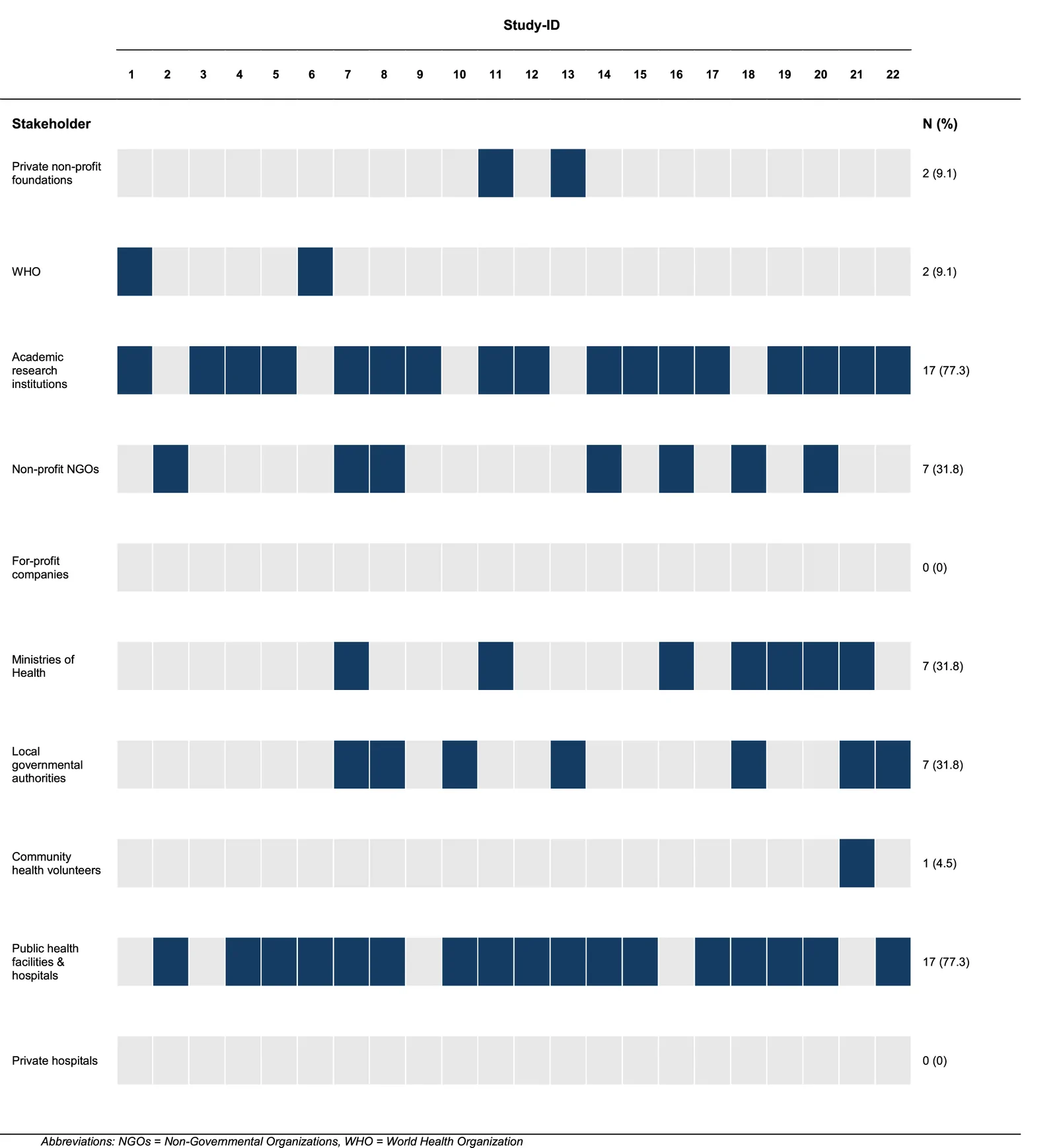
Stakeholders involved in implementing interventions.

Beyond descriptive mapping, clear functional distinctions emerged regarding stakeholder roles in leadership, funding, and implementation, as shown in Table 4. Government actors, particularly ministries of health [35,39,44,[46], [47], [48], [49]] and local governmental authorities [35,36,38,41,46,49,50], were central to stewardship functions. They primarily led policy implementation, coordinated health system activities, and supported integration and scale-up of interventions within routine maternal and neonatal care. Their role was especially prominent in district- or community-level interventions, where they ensured alignment with national strategies and facilitated institutionalization within public health systems. In several cases, ministries of health also engaged in selective co-development of interventions, particularly in adapting training programmes, quality improvement packages, and clinical innovations to local contexts [[46], [47], [48]]**.**

**Table 4:** Stakeholder analysis.

Stakeholder	Role (Stake)	Sector	N (%)	Example
Private non-profit foundations [39,41]	Project funding	Private non-profit/philanthropic sector	2 (9.1)	Supported development and implementation of interventions, including co-development of mHealth tools (e.g. Safe Delivery App) and quality improvement programmes (e.g. NEST), in collaboration with clinical staff in Rwanda and Burkina Faso
	Technical support/capacity building			
	Co-implementation			
	Co-development			
WHO [29,34]	Scale-up project funding	Multilateral public health organization (UN agency)	2 (9.1)	Provided global guidelines (e.g. KMC) and supported implementation and scale-up of evidence-based interventions
	Normative guidance			
	Research support			
	Intervention Implementation			
Academic research institutions [29,[31], [32], [33],[35], [36], [37],^39^,^40^,[42], [43], [44], [45],[47], [48], [49], [50]]	Research	Academic/research	17 (77.3)	Led clinical trials and implementation research, including evaluation of neonatal interventions, training programmes, and digital health tools
	Evaluation			
	Implementation support			
	Training			
Non-profit NGOs [30,35,36,42,44,46,48]	Implementation support	Non-governmental/non-profit sector	7 (31.8)	Delivered and co-implemented neonatal programmes, including training, quality improvement initiatives, and development of nutritional products (e.g., RUSF); supported programme planning, funding, and evaluation; contributed to mentorship and workforce training; developed and implemented medical devices and digital monitoring tools in collaboration with private-sector actors
	Technical and innovation support			
	Capacity building/training			
	Programme design			
	Evaluation support			
	Project funding			
Ministries of Health [35,39,44,[46], [47], [48], [49]]	Policy implementation	Public sector (national)	7 (31.8)	Supported implementation and scale-up, coordinated health system activities, and integrated programmes into routine care; contributed to adaptation of training, quality improvement packages, and clinical innovations
	Health system coordination			
	Scale-up support			
	Implementation support			
Local governmental authorities [[35], [36], [37],^41^,^46^,^49^,^50^]	Implementation support	Public sector (subnational)	7 (31.8)	Coordinated district-level implementation and supervised training and quality improvement activities
	Health system coordination			
	Quality improvement oversight			
Community health volunteers [49]	Implementation support	Community/public health system	1 (4,5)	Supported community linkage, newborn identification, and referral to health facilities
	Community linkage			
Public health facilities & hospitals [30,[32], [33], [34], [35], [36],[38], [39], [40], [41], [42], [43],[45], [46], [47], [48],^50^]	Quality improvement & clinical implementation	Public health system	17 (77.3)	Served as primary sites for intervention delivery, including clinical care, training and workforce provision, quality improvement activities, and clinical trials
	Service delivery			
	Research platform			
	Workforce provision			

Abbreviations: KMC = Kangaroo Mother Care, NEST = Neonatal Essential Survival Technology, NGOs = Non-Governmental Organizations, UN = United Nations, WHO = World Health Organization.

In contrast, implementation was largely anchored in public health facilities and hospitals, which served as the primary delivery platforms for interventions [30,[32], [33], [34], [35], [36],[38], [39], [40], [41], [42], [43],[45], [46], [47], [48],^50^]. These facilities provided clinical care, hosted training and quality improvement activities, functioned as research sites, and contributed to workforce capacity for service delivery and programme implementation.

Community-level actors, such as community health volunteers, played a limited but contextually important role in linking communities to facility-based care [49].

Non-profit NGOs emerged as key operational and intermediary actors, particularly in implementation support, capacity building, and programme delivery [30,35,36,42,44,46,48]. They frequently co-implemented neonatal health programmes, including clinical and management training, quality improvement initiatives, and the development of nutritional products such as ready-to-use supplementary food (RUSF) [30,35,36]. NGOs also contributed to programme planning, funding, monitoring, and evaluation, and played an important role in curriculum development, mentorship, and workforce training [30,35,36,44,46,48]. In several settings, NGOs worked in close collaboration with ministries of health, facilitating the implementation of newborn care bundles and hospital-based quality improvement interventions [46,47]. Additionally, some NGOs acted as technology and innovation partners, supporting the development and implementation of medical devices and digital monitoring tools in collaboration with private-sector-linked actors [42].

Academic research institutions played a dominant role in evidence generation and evaluation, leading clinical trials and implementation research across the majority of studies [29,31,37,43,45]. Their involvement often extended to implementation support, particularly in training and evaluation of intervention effectiveness, reflecting their central role in shaping the evidence base underpinning neonatal health interventions [29,33,35,36,[42], [43], [44], [45]].

Funding patterns indicated a reliance on external actors. International private non-profit foundations, such as those involved in the development of the Safe Delivery App and the NEST project, contributed financial resources, technical expertise, and co-development of interventions, including digital health tools and quality improvement programmes [39,41]. WHO contributed normative guidance, research support, and funding for scale-up of evidence-based interventions such as Kangaroo Mother Care [29,34]. Overall, funding and technical support were predominantly provided by external partners [40,41,44], while domestic actors were more strongly represented in coordination and implementation roles [47,49].

Clear patterns also emerged in partnership configurations. Government actors were involved in the majority of interventions, particularly those embedded in routine health systems. NGO involvement was most prominent in training, capacity building, and implementation support. Interventions involving technological innovation or more complex delivery models tended to rely on more diverse partnerships, combining academic institutions and private non-profit industry-linked actors, suggesting a need for additional technical expertise and resource mobilization [42].

Finally, the geographical distribution of organizations revealed an imbalance between domestic and external actors. Few organizations were based in low-income countries, with most stakeholders, particularly funders, academic institutions, and private foundations, headquartered in high-income countries [39,41,46,49]. This suggests that while implementation is largely locally embedded within public health systems, leadership in funding, innovation, and evidence generation remains predominantly externally driven.

### 3.4 Stakeholders involved in funding interventions

A clear distinction emerged between research funding, implementation or programme financing, and in-kind support.

Research funding for trial conduct and evaluation was reported in several randomized controlled trials and effectiveness studies. This included, for example, the immediate KMC trial [29] and the OMWaNA trial [45], which were embedded in externally funded global health research initiatives. Additional pre-post intervention studies and trials such as the *Helping Babies Breathe* evaluation [32,42,47] and other clinical effectiveness studies [31,37,38,43], were also conducted within externally supported research frameworks.

Implementation and programme financing were frequently linked to multilateral agencies and global health partnerships supporting scale-up or health system strengthening. This included WHO-supported post-discharge KMC scale-up [34], as well as quality improvement or training programmes embedded within national or district health systems, such as the LifeNet-funded clinical and management training programme [30], the Saving Lives at Birth-supported *All Babies Count* project [46], Chiesi Foundation-funded *Neonatal Essential Survival Technology* programme [41].

In-kind contributions and commodity support were reported in several studies, particularly within quality improvement and neonatal care strengthening interventions. These included donations of equipment, consumables, and supplies from non-profit organizations and implementing partners, as well as technical and training support provided alongside service delivery interventions [34,40,44].

Overall, many studies did not report detailed funding information or only provided general statements. This limited transparency restricts the ability to clearly differentiate between funding for evidence generation and funding for intervention delivery.

## 4 Discussion

### 4.1 Main findings

Various interventions targeting prematurity, birth asphyxia, and neonatal infections have been implemented in LICs since the adoption of SDG 3.2. These interventions encompassed quality of neonatal care improvement initiatives, neonatal resuscitation training, KMC implementation strategies, pharmaceutical and nutritional interventions, mHealth interventions, and thermal and respiratory support.

Across countries, a limited set of recurring intervention approaches predominated, with facility-based strategies (e.g., KMC, neonatal resuscitation training, and quality improvement) most common in East Africa, while West African studies more frequently focused on biomedical and preventive interventions, and multi-country studies emphasized standardized clinical approaches.

These findings suggest that intervention implementation in LICs is not primarily shaped by country-specific needs but rather by the transfer and scaling of a limited set of established intervention models. The dominance of facility-based approaches, particularly KMC, neonatal resuscitation training, and quality improvement packages, reflects a focus on interventions that are considered scalable and evidence-based, rather than contextually tailored innovations. At the same time, the concentration of biomedical interventions in West African settings may indicate a stronger emphasis on clinical or preventive strategies in these contexts, although this pattern may also reflect differences in research priorities. The predominance of facility-level interventions further suggests that efforts are largely concentrated on strengthening service delivery within health facilities, while comparatively limited attention is given to community- or system-level approaches, which may be critical for improving access, early identification, and continuity of care.

Most of the included interventions aimed at improving the quality of care, addressing both structural and process-related dimensions, supporting existing evidence that poor quality of care is a key determinant of neonatal mortality in LICs [51]. The provision of high-quality, accessible, and financially affordable neonatal care requires functional health facilities, skilled workforce, essential medical supplies and equipment, effective data management, and continuity of care [52], core elements of Universal Health Coverage [53].

Although no formal critical appraisal was conducted in line with the scoping review methodology, an overview of study designs is provided to characterize the evidence base. This indicates that a substantial proportion of studies are randomized controlled trials, which represent a comparatively high level of methodological rigor. In addition, several included studies evaluated well-established interventions such as KMC, Helping Babies Breathe, and the All Babies Count programme, for which a substantial existing evidence base on effectiveness is already available [[54], [55], [56], [57], [58]].

The most frequently reported collaborators were public health facilities, international academic institutions, non-profit NGOs, ministries of health, and local governmental authorities and stakeholders. Public health facilities and international academic research institutions appeared most often, likely because the reviewed interventions focused on intrapartum, immediate newborn, and postpartum care. Antenatal care, often provided in community settings and through community health workers, are less reported in the included interventions. Nevertheless, the provision of high-quality antenatal care is also crucial for reducing neonatal mortality by enabling the early prevention, detection, and treatment of risk factors during pregnancy, and for advancing progress towards the SDG targets [52,59]. The observed stakeholder patterns further highlight a division of roles within implementation partnerships. Government actors, particularly ministries of health and local governmental authorities, appear central to coordination at the health system level, integration into routine care, and scale-up of interventions. In contrast, NGOs primarily contribute to implementation support, training, and capacity building, often acting as intermediaries between global evidence and local practice. Academic institutions play a dominant role in generating and evaluating evidence, reflecting their strong presence across study designs. Interventions involving technological innovation or more complex delivery models tend to rely on more diverse partnerships, including private non-profit foundations and private-sector-linked actors, suggesting that such approaches require additional technical expertise and resource mobilization. Overall, these patterns indicate complementary but unevenly distributed stakeholder roles, with service delivery largely anchored in public health facilities, while external actors contribute substantially to implementation support, innovation, and evidence generation, with potential implications for sustainability and local ownership of interventions. Most studies included in this review offered limited information on stakeholder involvement. However, multi-stakeholder partnerships can catalyse capacity-building for neonatal health interventions in LICs through training, mentorship, knowledge exchange, procurement and systems support [52,59]. However, multi-stakeholder initiatives have also highlighted challenges such as unclear governance, power imbalances between local and international partners, short project timelines [60]. Equitable collaborative frameworks, formalised in memoranda of understanding, co-created objectives, transparent decision making, realistic timelines and long-term sustainability strategies are critical. Meaningful engagement of local stakeholders, including frontline staff and community representatives, improves contextual fit and ownership.

This review found that few organizations were based in LICs, with most headquartered in high-income countries. This is consistent with findings from Moon et al. (2024) who performed a landscape analysis of key stakeholders involved in addressing preterm birth [15]. The authors identified interventions by mapping organizations directly. In contrast, the review presented here identified multi-stakeholder partnerships by first mapping interventions implemented in LICs and then tracing the stakeholders involved. Therefore, local governmental (health) authorities and stakeholders could be better identified. Despite the different methodological approach, there was considerable overlap in the stakeholder categories identified including knowledge production organizations, funders, legislation and advocacy organizations, and implementers [15].

Few studies identified which stakeholders or organizations contributed to intervention funding [30,34,40,41,44,46]. Intransparency and underreporting of financing pathways, donor contributions, domestic resource allocation and partner roles constrains assessment of how neonatal health interventions are financed, sustained and scaled in LIC.

One study on KMC implementation across several LICs showed that while initial financing was supported by donors and partners, national governments gradually took ownership by allocating domestic funds to sustain and scale services [61]. This urges the need for improved action and implementation research in LICs to generate practice-relevant evidence on the scalability, transferability and equity implications of neonatal health programmes, promoting more evidence-based decision-making by governments, funders and implementing partners.

The absence of studies from low-income countries outside sub-Saharan Africa, as identified in this scoping review, suggests a substantial geographical evidence gap in maternal and neonatal health intervention research. However, this gap may partly reflect the fragile and conflict-affected contexts of these countries, where protracted humanitarian crises, political instability, and weakened health systems can constrain both the implementation of interventions and the conduct and publication of research. For instance, in Syria, the partial breakdown of healthcare infrastructure combined with delays in humanitarian coordination due to ongoing conflict has impacted delivery and accessibility of neonatal care services [62]. Conflict-affected settings often face higher neonatal mortality rates and lower coverage of essential maternal and newborn interventions [63]. The implementation of interventions in conflict-affected regions is likely to encounter substantial challenges due to insecurity, displacement, disrupted health systems, and limited access to affected populations [64]. Consequently, the current evidence base may be shaped not only by research priorities but also by feasibility and access limitations in these settings.

Moreover, the review period (2015–2025) coincided with the COVID-19 pandemic, which had impacts on health systems globally. Some studies included in this review were affected by pandemic-related interruptions, delays or hygiene regulations [32,42,43,45,48]. Evidence from other LMICs suggests that the COVID-19 pandemic and associated mitigation measures may have impacted the provision of neonatal health services [65,66], for example through a reduction of antenatal care visits [65,67].

### 4.2 Strengths and limitations

This scoping review provides the first comprehensive mapping of post-2015 interventions in LICs targeting the three leading causes of neonatal mortality, aiming to assess how the global goals have translated into practice. Limiting the review to LICs enhances applicability to the most resource-constrained settings. In addition, this review describes multi-stakeholder partnerships involved in the financing and implementation of these health interventions, exploring how governments, non-governmental organizations, donors, and other partners collaborate to improve neonatal health. As added value, this review not only examines the scope and characteristics of these partnerships but also the perceived challenges in sustainable implementation and financing. In doing so, the review seeks to critically discuss levels of reciprocal knowledge exchange, shared decision-making and equitable collaboration in multi-stakeholder partnerships to inform future research on partnership models for improving neonatal health in LICs.

Limitations include the restriction to English-language publications, which may have introduced language bias by potentially excluding relevant studies published in other languages and thereby affecting the comprehensiveness of the evidence base. Furthermore, due to the constraints of a six-month master's thesis, full dual independent screening of all full-text articles was not feasible; instead, a 20 % sample was independently verified, which may have introduced selection bias despite efforts to ensure accuracy and consistency. The search strategy, including the use of Google Scholar, may also have introduced selection bias due to its algorithm-driven and less reproducible nature compared to database-only searches. In addition, no formal quality appraisal of included studies was conducted, which limits the ability to assess the robustness of the underlying evidence. Additional limitations include the potential omission of relevant interventions addressing the main causes of neonatal mortality but not reporting neonatal mortality as an outcome, as well as the underrepresentation of grey literature, which may contribute to publication bias by favouring peer-reviewed studies. Furthermore, stakeholder involvement and funding information were frequently underreported in the included studies, which may have limited the completeness and accuracy of the mapping of multi-stakeholder partnerships and financing structures. The heterogeneity of included interventions may also limit comparability across studies, and thematic grouping of interventions may be subject to misclassification bias due to variability and ambiguity in how interventions were described and reported.

### 4.3 Conclusion

Since the adoption of SDG 3.2 in 2015, neonatal health interventions in low-income countries have predominantly focused on a limited set of facility-based approaches, particularly Kangaroo Mother Care, neonatal resuscitation training, and quality improvement packages. These interventions are largely embedded within routine care in public health facilities, reflecting a strong emphasis on strengthening service delivery at the facility level.

Implementation is typically driven by multi-stakeholder partnerships with differentiated roles. Domestic actors, including Ministries of Health and public health facilities, are central to coordination and service delivery, while external partners, such as academic institutions, NGOs, and private foundations primarily contribute funding, technical support, and evidence generation. Although these complementary roles facilitate implementation, they also highlight structural imbalances in global health partnerships.

Intervention types and partnership configurations show regional clustering, and most interventions remain facility-based, with limited engagement at the community or district level.

Furthermore, inconsistent reporting of stakeholder roles, particularly in relation to funding, governance and implementation responsibilities limits assessment of sustainability. Sustained investment is therefore critical to advance maternal and neonatal health beyond the duration of externally funded interventions. This is especially important in an increasingly fragmented global health landscape, where declining international funding and weakening long-term commitments undermine progress towards the SDGs. Future efforts should strengthen transparency and community linkages, promote domestic ownership and sustainable financing mechanisms, and facilitate integration into national health systems. These measures are critical to safeguarding sexual and reproductive health and rights and sustaining progress in reducing neonatal mortality.

## Ethical statement

This scoping review uses existing published studies. Ethical approval from an ethics committee was therefore not required.

## Authors’ contribution

MR conceptualized and designed the study. MR and MP collected the data, while MR analysed the data. MR and VF drafted the initial manuscript. VF and AK supervised the project, reviewed, and edited the manuscript. All authors contributed to the article and approved the submitted version.

## Funding

No funding was received for this study.

## Declaration of competing interest

The authors declare no financial or non-financial competing interests that could have influenced the work reported in this review article:

Reducing neonatal mortality in low-income countries: A scoping review of health interventions and multi-stakeholder partnerships implemented since the adoption of Sustainable Development Goal 3.2.
