# Supplementary material for: Health interventions and stakeholder roles in neonatal mortality reduction within low-income countries: A scoping review
Source: Public Health Pract (Oxf). 2026 Aug 21;12:100847. doi: 10.1016/j.puhip.2026.100847 (PMC13640466; doi:10.1016/j.puhip.2026.100847)
Supplement: Supplementary file 2 [file mmc2.docx]

**A1 Peer-reviewed databases**

**Medline Ovid**

| Search | Terms | Hits |
| --- | --- | --- |
| 1 | Infant, Newborn/ | 696,147 |
| 2 | newborn?.ab,kf,ti. | 209,462 |
| 3 | neonate?.ab,kf,ti. | 117,715 |
| 4 | Infant, Premature/ | 64,866 |
| 5 | Preterm infant?.ab,kf,ti. | 36,188 |
| 6 | prematur*.ab,kf,ti. | 188,241 |
| 7 | Preterm neonate?.ab,kf,ti. | 7,179 |
| 8 | Infant, Low Birth Weight/ | 20,819 |
| 9 | Low birth weight infant?.ab,kf,ti. | 9,881 |
| 10 | Very low birth weight infant?.ab,kf,ti. | 4,761 |
| 11 | Extremely low birth weight infant?.ab,kf,ti. | 1,603 |
| 12 | 1 or 2 or 3 or 4 or 5 or 6 or 7 or 8 or 9 or 10 or 11 | 936,805 |
| 13 | health intervention?.ab,kf,ti. | 27,320 |
| 14 | intervention?.ab,kf,ti. | 1,482,029 |
| 15 | Pregnancy/ | 1,033,796 |
| 16 | Pregnanc*.ab,kf,ti. | 555,169 |
| 17 | Maternal Health Services/ | 17,377 |
| 18 | Maternal health service?.ab,kf,ti. | 2,806 |
| 19 | Midwifery/ | 22,226 |
| 20 | midwifery.ab,kf,ti. | 13,987 |
| 21 | birth attendant?.ab,kf,ti. | 2,915 |
| 22 | Postnatal Care/ | 6,703 |
| 23 | postnatal care.ab,kf,ti. | 3,306 |
| 24 | Prenatal Care/ | 34,319 |
| 25 | prenatal care.ab,kf,ti. | 14,540 |
| 26 | intrapartum care.ab,kf,ti. | 1,145 |
| 27 | antenatal care.ab,kf,ti. | 15,263 |
| 28 | neonatal care.ab,kf,ti. | 5,421 |
| 29 | Continuity of Patient Care/ | 21,237 |
| 30 | continuum of care.ab,kf,ti. | 3,848 |
| 31 | Quality Improvement/ | 35,632 |
| 32 | quality improvement?.ab,kf,ti. | 63,126 |
| 33 | quality.ab,kf,ti. | 1,596,109 |
| 34 | Delivery of Health Care/ | 124,597 |
| 35 | health care deliver*.ab,kf,ti. | 13,562 |
| 36 | Program Evaluation/ | 68,751 |
| 37 | evaluation.ab,kf,ti. | 1,610,498 |
| 38 | sustainabilit*.ab,kf,ti. | 59,967 |
| 39 | Primary Prevention/ | 20,419 |
| 40 | Primary prevention.ab,kf,ti. | 25,370 |
| 41 | Health Promotion/ | 84,661 |
| 42 | health promotion.ab,kf,ti. | 46,988 |
| 43 | Primary Health Care/ | 97,548 |
| 44 | primary care.ab,kf,ti. | 160,244 |
| 45 | primary health care.ab,kf,ti. | 37,459 |
| 46 | Public Health/ | 100,324 |
| 47 | public health.ab,kf,ti. | 429,471 |
| 48 | community health.ab,kf,ti. | 36,341 |
| 49 | Health Education/ | 64,886 |
| 50 | health education.ab,kf,ti. | 47,914 |
| 51 | Disease Management/ | 44,018 |
| 52 | disease management.ab,kf,ti. | 27,766 |
| 53 | Therapeutics/ | 8,587 |
| 54 | therapeutic?.ab,kf,ti. | 1,618,246 |
| 55 | treatment?.ab,kf,ti. | 6,029,520 |
| 56 | therap*.ab,kf,ti. | 3,986,493 |
| 57 | 13 or 14 or 15 or 16 or 17 or 18 or 19 or 20 or 21 or 22 or 23 or 24 or 25 or 26 or 27 or 28 or 29 or 30 or 31 or 32 or 33 or 34 or 35 or 36 or 37 or 38 or 39 or 40 or 41 or 42 or 43 or 44 or 45 or 46 or 47 or 48 or 49 or 50 or 51 or 52 or 53 or 54 or 55 or 56 | 12,409,264 |
| 58 | Infant Mortality/ | 30,643 |
| 59 | infant mortality.ab,kf,ti. | 15,119 |
| 60 | newborn mortality.ab,kf,ti. | 515 |
| 61 | neonatal mortality.ab,kf,ti. | 10,310 |
| 62 | Perinatal Mortality/ | 2,566 |
| 63 | perinatal mortality.ab,kf,ti. | 11,215 |
| 64 | 58 or 59 or 60 or 61 or 62 or 63 | 50,025 |
| 65 | Afghanistan/ | 4,005 |
| 66 | Afghanistan.ab,kf,ti. | 7,760 |
| 67 | Burkina Faso/ | 4,311 |
| 68 | Burkina Faso.ab,kf,ti. | 5,728 |
| 69 | Burundi/ | 745 |
| 70 | Burundi.ab,kf,ti. | 1,157 |
| 71 | Central African Republic/ | 870 |
| 72 | Central African Republic.ab,kf,ti. | 1,259 |
| 73 | Chad/ | 876 |
| 74 | Chad.ab,kf,ti. | 1,685 |
| 75 | Democratic Republic of the Congo/ | 5,413 |
| 76 | Democratic Republic of the Congo.ab,kf,ti. | 4,883 |
| 77 | Democratic People's Republic of Korea/ | 322 |
| 78 | Democratic People's Republic of Korea.ab,kf,ti. | 148 |
| 79 | DPRK.ab,kf,ti. | 53 |
| 80 | North Korea.ab,kf,ti. | 517 |
| 81 | Eritrea/ | 451 |
| 82 | Eritrea.ab,kf,ti. | 800 |
| 83 | Ethiopia/ | 22,923 |
| 84 | Ethiopia.ab,kf,ti. | 34,216 |
| 85 | Gambia/ | 2,818 |
| 86 | Gambia.ab,kf,ti. | 3,025 |
| 87 | Guinea-Bissau/ | 1,064 |
| 88 | Guinea-Bissau.ab,kf,ti. | 1,204 |
| 89 | Liberia/ | 1,468 |
| 90 | Liberia.ab,kf,ti. | 2,102 |
| 91 | Madagascar/ | 4,107 |
| 92 | Madagascar.ab,kf,ti. | 6,243 |
| 93 | Malawi/ | 7,269 |
| 94 | Malawi.ab,kf,ti. | 9,997 |
| 95 | Mali/ | 2,874 |
| 96 | Mali.ab,kf,ti. | 4,938 |
| 97 | Mozambique/ | 3,303 |
| 98 | Mozambique.ab,kf,ti. | 5,133 |
| 99 | Niger/ | 1,522 |
| 100 | Niger.ab,kf,ti. | 15,891 |
| 101 | Rwanda/ | 3,508 |
| 102 | Rwanda.ab,kf,ti. | 4,801 |
| 103 | Sierra Leone/ | 2,110 |
| 104 | Sierra Leone.ab,kf,ti. | 3,260 |
| 105 | Somalia/ | 2,040 |
| 106 | Somalia.ab,kf,ti. | 2,138 |
| 107 | South Sudan/ | 330 |
| 108 | South Sudan.ab,kf,ti. | 947 |
| 109 | Sudan/ | 5,413 |
| 110 | Sudan.ab,kf,ti. | 10,645 |
| 111 | Syria/ | 2,897 |
| 112 | Syria.ab,kf,ti. | 3,464 |
| 113 | Togo/ | 1,349 |
| 114 | Togo.ab,kf,ti. | 1,997 |
| 115 | Uganda/ | 16,908 |
| 116 | Uganda.ab,kf,ti. | 21,833 |
| 117 | Yemen/ | 1,666 |
| 118 | Yemen.ab,kf,ti. | 2,568 |
| 119 | Developing Countries/ | 83,558 |
| 120 | developing countr*.ab,kf,ti. | 107,803 |
| 121 | low income countr*.ab,kf,ti. | 11,564 |
| 122 | LIC?.ab,kf,ti. | 16,600 |
| 123 | 65 or 66 or 67 or 68 or 69 or 70 or 71 or 72 or 73 or 74 or 75 or 76 or 77 or 78 or 79 or 80 or 81 or 82 or 83 or 84 or 85 or 86 or 87 or 88 or 89 or 90 or 91 or 92 or 93 or 94 or 95 or 96 or 97 or 98 or 99 or 100 or 101 or 102 or 103 or 104 or 105 or 106 or 107 or 108 or 109 or 110 or 111 or 112 or 113 or 114 or 115 or 116 or 117 or 118 or 119 or 120 or 121 or 122 | 325,546 |
| 124 | 12 and 57 and 64 and 123 | 3,734 |
| 125 | limit 124 to yr="2015 -Current" | 1,539 |

**Embase Ovid**

| Search | Terms | Hits |
| --- | --- | --- |
| 1 | newborn/ | 734,942 |
| 2 | newborn?.ab,kf,ti. | 282,216 |
| 3 | neonate?.ab,kf,ti. | 165,837 |
| 4 | prematurity/ | 156,698 |
| 5 | Preterm infant?.ab,kf,ti. | 46,931 |
| 6 | prematur*.ab,kf,ti. | 275,088 |
| 7 | Preterm neonate?.ab,kf,ti. | 9,846 |
| 8 | low birth weight/ | 47,698 |
| 9 | Low birth weight infant?.ab,kf,ti. | 12,437 |
| 10 | Very low birth weight infant?.ab,kf,ti. | 5,875 |
| 11 | Extremely low birth weight infant?.ab,kf,ti. | 2,028 |
| 12 | 1 or 2 or 3 or 4 or 5 or 6 or 7 or 8 or 9 or 10 or 11 | 1,135,749 |
| 13 | health intervention?.ab,kf,ti. | 30,312 |
| 14 | intervention?.ab,kf,ti. | 2,021,308 |
| 15 | pregnancy/ | 879,601 |
| 16 | Pregnanc*.ab,kf,ti. | 781,839 |
| 17 | maternal health service/ | 3,564 |
| 18 | Maternal health service?.ab,kf,ti. | 1,882 |
| 19 | midwife/ | 34,030 |
| 20 | midwifery.ab,kf,ti. | 15,344 |
| 21 | birth attendant?.ab,kf,ti. | 3,079 |
| 22 | postnatal care/ | 10,174 |
| 23 | postnatal care.ab,kf,ti. | 3,838 |
| 24 | prenatal care/ | 58,876 |
| 25 | prenatal care.ab,kf,ti. | 17,631 |
| 26 | intrapartum care.ab,kf,ti. | 1,463 |
| 27 | antenatal care.ab,kf,ti. | 18,762 |
| 28 | neonatal care.ab,kf,ti. | 7,398 |
| 29 | continuum of care.ab,kf,ti. | 5,305 |
| 30 | quality improvement?.ab,kf,ti. | 98,974 |
| 31 | quality.ab,kf,ti. | 2,194,763 |
| 32 | health care delivery/ | 222,986 |
| 33 | health care deliver*.ab,kf,ti. | 16,978 |
| 34 | program evaluation/ | 20,431 |
| 35 | evaluation.ab,kf,ti. | 2,304,062 |
| 36 | sustainabilit*.ab,kf,ti. | 64,484 |
| 37 | primary prevention/ | 50027 |
| 38 | Primary prevention.ab,kf,ti. | 38411 |
| 39 | health promotion/ | 117,948 |
| 40 | health promotion.ab,kf,ti. | 55,085 |
| 41 | primary health care/ | 84,617 |
| 42 | primary care.ab,kf,ti. | 219,274 |
| 43 | primary health care.ab,kf,ti. | 41,981 |
| 44 | public health/ | 273,402 |
| 45 | public health.ab,kf,ti. | 481,803 |
| 46 | community health.ab,kf,ti. | 44,255 |
| 47 | health education/ | 124,115 |
| 48 | health education.ab,kf,ti. | 57,503 |
| 49 | disease management/ | 32,614 |
| 50 | disease management.ab,kf,ti. | 38,809 |
| 51 | therapy/ | 1,506,905 |
| 52 | therapeutic?.ab,kf,ti. | 2,232,812 |
| 53 | treatment?.ab,kf,ti. | 8,765,350 |
| 54 | therap*.ab,kf,ti. | 5,812,203 |
| 55 | 13 or 14 or 15 or 16 or 17 or 18 or 19 or 20 or 21 or 22 or 23 or 24 or 25 or 26 or 27 or 28 or 29 or 30 or 31 or 32 or 33 or 34 or 35 or 36 or 37 or 38 or 39 or 40 or 41 or 42 or 43 or 44 or 45 or 46 or 47 or 48 or 49 or 50 or 51 or 52 or 53 or 54 | 17,478,818 |
| 56 | infant mortality/ | 29,064 |
| 57 | infant mortality.ab,kf,ti. | 14,157 |
| 58 | newborn mortality.ab,kf,ti. | 662 |
| 59 | neonatal mortality.ab,kf,ti. | 13,543 |
| 60 | perinatal mortality/ | 16,485 |
| 61 | perinatal mortality.ab,kf,ti. | 16,400 |
| 62 | 56 or 57 or 58 or 59 or 60 or 61 | 64,392 |
| 63 | Afghanistan/ | 7,797 |
| 64 | Afghanistan.ab,kf,ti. | 9,259 |
| 65 | Burkina Faso/ | 6,097 |
| 66 | Burkina Faso.ab,kf,ti. | 6,749 |
| 67 | Burundi/ | 1,225 |
| 68 | Burundi.ab,kf,ti. | 1,280 |
| 69 | Central African Republic/ | 1,155 |
| 70 | Central African Republic.ab,kf,ti. | 1,395 |
| 71 | Chad/ | 1,246 |
| 72 | Chad.ab,kf,ti. | 2,190 |
| 73 | Democratic Republic Congo/ | 6,061 |
| 74 | Democratic Republic of the Congo.ab,kf,ti. | 5,790 |
| 75 | North Korea/ | 759 |
| 76 | Democratic People's Republic of Korea.ab,kf,ti. | 128 |
| 77 | DPRK.ab,kf,ti. | 55 |
| 78 | North Korea.ab,kf,ti. | 597 |
| 79 | Eritrea/ | 815 |
| 80 | Eritrea.ab,kf,ti. | 992 |
| 81 | Ethiopia/ | 32,896 |
| 82 | Ethiopia.ab,kf,ti. | 34,495 |
| 83 | Gambia/ | 3,568 |
| 84 | Gambia.ab,kf,ti. | 3,484 |
| 85 | Guinea-Bissau/ | 1,350 |
| 86 | Guinea-Bissau.ab,kf,ti. | 1,394 |
| 87 | Liberia/ | 2,359 |
| 88 | Liberia.ab,kf,ti. | 2,475 |
| 89 | Madagascar/ | 5,831 |
| 90 | Madagascar.ab,kf,ti. | 7,067 |
| 91 | Malawi/ | 10,779 |
| 92 | Malawi.ab,kf,ti. | 11,660 |
| 93 | Mali/ | 4.656 |
| 94 | Mali.ab,kf,ti. | 5,961 |
| 95 | Mozambique/ | 5,449 |
| 96 | Mozambique.ab,kf,ti. | 5,993 |
| 97 | Niger/ | 3,184 |
| 98 | Niger.ab,kf,ti. | 21,070 |
| 99 | Rwanda/ | 5,622 |
| 100 | Rwanda.ab,kf,ti. | 5,695 |
| 101 | Sierra Leone/ | 3,433 |
| 102 | Sierra Leone.ab,kf,ti. | 3,739 |
| 103 | Somalia/ | 2,777 |
| 104 | Somalia.ab,kf,ti. | 2,334 |
| 105 | South Sudan/ | 660 |
| 106 | South Sudan.ab,kf,ti. | 1,075 |
| 107 | Sudan/ | 9,262 |
| 108 | Sudan.ab,kf,ti. | 14,019 |
| 109 | Syrian Arab Republic/ | 4,153 |
| 110 | Syria.ab,kf,ti. | 4,109 |
| 111 | Togo/ | 1,970 |
| 112 | Togo.ab,kf,ti. | 2,331 |
| 113 | Uganda/ | 26,285 |
| 114 | Uganda.ab,kf,ti. | 26,487 |
| 115 | Yemen/ | 2,762 |
| 116 | Yemen.ab,kf,ti. | 2,961 |
| 117 | developing country/ | 104,830 |
| 118 | developing countr*.ab,kf,ti. | 104,411 |
| 119 | low income countr*.ab,kf,ti. | 14,041 |
| 120 | LIC?.ab,kf,ti. | 20,685 |
| 121 | 63 or 64 or 65 or 66 or 67 or 68 or 69 or 70 or 71 or 72 or 73 or 74 or 75 or 76 or 77 or 78 or 79 or 80 or 81 or 82 or 83 or 84 or 85 or 86 or 87 or 88 or 89 or 90 or 91 or 92 or 93 or 94 or 95 or 96 or 97 or 98 or 99 or 100 or 101 or 102 or 103 or 104 or 105 or 106 or 107 or 108 or 109 or 110 or 111 or 112 or 113 or 114 or 115 or 116 or 117 or 118 or 119 or 120 | 375,321 |
| 122 | 12 and 55 and 62 and 121 | 2,657 |
| 123 | limit 122 to yr="2015 -Current" | 1,353 |

**CINAHL**

| Search | Terms | Hits |
| --- | --- | --- |
| 1 | (MH "Infant, Newborn") | 146,835 |
| 2 | (TI newborn# OR AB newborn#) | 37,871 |
| 3 | (TI neonate# OR AB neonate#) | 28,220 |
| 4 | (SU Infant, Newborn) | 147,536 |
| 5 | (MH "Infant, Premature") | 27,596 |
| 6 | (TI Preterm infant# OR AB Preterm infant#) | 17,511 |
| 7 | (TI prematur* OR AB prematur*) | 38,878 |
| 8 | (TI Preterm neonate# OR AB Preterm neonate#) | 3,312 |
| 9 | (SU Infant, Premature) | 29,093 |
| 10 | (MH "Infant, Low Birth Weight") | 8,141 |
| 11 | (TI Low birth weight infant# OR AB Low birth weight infant#) | 5,140 |
| 12 | (TI Very low birth weight infant# OR AB Very low birth weight infant#) | 2,546 |
| 13 | (TI Extremely low birth weight infant# OR AB Extremely low birth weight infant#) | 954 |
| 14 | (SU Infant, Low Birth Weight) | 13,609 |
| 15 | (S1 OR S2 OR S3 OR S4 OR S5 OR S6 OR S7 OR S8 OR S9 OR S10 OR S11 OR S12 OR S13 OR S14) | 208,120 |
| 16 | (MH "Nursing Interventions") | 11,157 |
| 17 | (TI health intervention# OR AB health intervention#) | 48,100 |
| 18 | (TI intervention# OR AB intervention#) | 578,464 |
| 19 | (SU Nursing Interventions) | 11,890 |
| 20 | (MH "Pregnancy") | 229,330 |
| 21 | (TI Pregnanc* OR AB Pregnanc*) | 146,822 |
| 22 | (SU Pregnancy) | 231,636 |
| 23 | (TI "Maternal health service#" OR AB "Maternal health service#") | 763 |
| 24 | (SU Maternal Health Services) | 12,538 |
| 25 | (MH "Midwifery") | 18,015 |
| 26 | (TI midwifery OR AB midwifery) | 15,872 |
| 27 | (TI "birth attendant" OR AB "birth attendant") | 417 |
| 28 | (SU Midwifery) | 33,790 |
| 29 | (MH "Postnatal Care") | 7,032 |
| 30 | (TI "postnatal care" OR AB "postnatal care") | 1,625 |
| 31 | (SU Postnatal Care) | 7,032 |
| 32 | (MH "Prenatal Care") | 20,934 |
| 33 | (TI "prenatal care" OR AB "prenatal care") | 6,330 |
| 34 | (TI "intrapartum care" OR AB "intrapartum care") | 656 |
| 35 | (TI "antenatal care" OR AB "antenatal care") | 6,128 |
| 36 | (TI "neonatal care" OR AB "neonatal care") | 2,464 |
| 37 | (SU Prenatal Care) | 20,935 |
| 38 | (MH "Continuity of Patient Care") | 17,994 |
| 39 | (TI "continuum of care" OR AB "continuum of care") | 2,437 |
| 40 | (SU Continuity of Patient Care) | 17,994 |
| 41 | (MH "Quality Improvement") | 75,137 |
| 42 | (TI "quality improvement#" OR AB "quality improvement#") | 30,156 |
| 43 | (TI quality OR AB quality) | 484,982 |
| 44 | (SU Quality Improvement) | 75,166 |
| 45 | (MH "Health Care Delivery") | 70,473 |
| 46 | (TI "health care deliver*" OR AB "health care deliver*") | 6,195 |
| 47 | (SU Health Care Delivery) | 86,654 |
| 48 | (MH "Program Evaluation") | 53,728 |
| 49 | (TI evaluation OR AB evaluation) | 374,238 |
| 50 | (TI sustainabilit* OR AB sustainabilit*) | 13,691 |
| 51 | (SU Program Evaluation) | 57,059 |
| 52 | (MH "Preventive Health Care") | 24,475 |
| 53 | (TI "Primary prevention" OR AB "Primary prevention") | 8,144 |
| 54 | (SU Preventive Health Care) | 24,475 |
| 55 | (MH "Health Promotion") | 84,868 |
| 56 | (TI "health promotion" OR AB "health promotion") | 26,225 |
| 57 | (SU Health Promotion) | 85,167 |
| 58 | (MH "Primary Health Care") | 76,950 |
| 59 | (TI "primary care" OR AB "primary care") | 86,463 |
| 60 | (SU Primary Health Care) | 76,950 |
| 61 | (MH "Public Health") | 66,026 |
| 62 | (TI "public health" OR AB "public health") | 120,421 |
| 63 | (TI "community health" OR AB "community health") | 18,761 |
| 64 | (SU Public Health) | 74,368 |
| 65 | (MH "Health Education") | 33,629 |
| 66 | (TI "health education" OR AB "health education") | 20,843 |
| 67 | (SU Health Education) | 71,555 |
| 68 | (MH "Disease Management") | 26,787 |
| 69 | (TI "disease management" OR AB "disease management") | 8,999 |
| 70 | (SU Disease Management) | 26,787 |
| 71 | (MH Therapeutics) | 4,807 |
| 72 | (TI therapeutic# OR AB therapeutic#) | 205,754 |
| 73 | (TI treatment# OR AB treatment#) | 1,087,135 |
| 74 | (TI therap* OR AB therap*) | 738,401 |
| 75 | (SU Therapeutics) | 637,339 |
| 76 | (S16 OR S17 OR S18 OR S19 OR S20 OR S21 OR S22 OR S23 OR S24 OR S25 OR S26 OR S27 OR S28 OR S29 OR S30 OR S31 OR S32 OR S33 OR S34 OR S35 OR S36 OR S37 OR S38 OR S39 OR S40 OR S41 OR S42 OR S43 OR S44 OR S45 OR S46 OR S47 OR S48 OR S49 OR S50 OR S51 OR S52 OR S53 OR S54 OR S55 OR S56 OR S57 OR S58 OR S59 OR S60 OR S61 OR S62 OR S63 OR S64 OR S65 OR S66 OR S67 OR S68 OR S69 OR S70 OR S71 OR S72 OR S73 OR S74 OR S75) | 3,269,574 |
| 77 | (MH "Infant Mortality") | 10,652 |
| 78 | (TI "newborn mortality" OR AB "newborn mortality") | 210 |
| 79 | (SU Infant Mortality) | 11,067 |
| 80 | (MH "Perinatal Death") | 9,404 |
| 81 | (TI "perinatal mortality" OR AB "perinatal mortality") | 2,268 |
| 82 | (TI "infant mortality" OR AB "infant mortality") | 3,325 |
| 83 | (SU Perinatal Death) | 9,404 |
| 84 | (S77 OR S78 OR S79 OR S80 OR S81 OR S82 OR S83) | 21,752 |
| 85 | (MH "Afghanistan") | 2,814 |
| 86 | (TI Afghanistan OR AB Afghanistan) | 3,471 |
| 87 | (SU Afghanistan) | 2,818 |
| 88 | (MH "Burkina Faso") | 1,001 |
| 89 | (TI "Burkina Faso" OR AB "Burkina Faso") | 1,392 |
| 90 | (SU Burkina Faso) | 1,001 |
| 91 | (MH "Burundi") | 171 |
| 92 | (TI Burundi OR AB Burundi) | 257 |
| 93 | (SU Burundi) | 171 |
| 94 | (MH "Central African Republic") | 129 |
| 95 | (TI "Central African Republic" OR AB "Central African Republic") | 183 |
| 96 | (SU Central African Republic) | 129 |
| 97 | (MH "Chad") | 166 |
| 98 | (TI Chad OR AB Chad) | 479 |
| 99 | (SU Chad) | 166 |
| 100 | (MH "Democratic Republic of the Congo") | 1,128 |
| 101 | (TI "Democratic Republic of the Congo" OR AB "Democratic Republic of the Congo") | 607 |
| 102 | (SU Democratic Republic of the Congo) | 1,132 |
| 103 | (MH "North Korea") | 214 |
| 104 | (TI "North Korea" OR AB "North Korea") | 144 |
| 105 | (TI DPRK OR AB DPRK) | 11 |
| 106 | (TI "Democratic People's Republic of Korea" OR AB "Democratic People's Republic of Korea") | 21 |
| 107 | (SU North Korea) | 215 |
| 108 | (MH "Eritrea") | 163 |
| 109 | (TI Eritrea OR AB Eritrea) | 204 |
| 110 | (SU Eritrea) | 163 |
| 111 | (MH "Ethiopia") | 7,437 |
| 112 | (TI Ethiopia OR AB Ethiopia) | 9,114 |
| 113 | (SU Ethiopia) | 7,453 |
| 114 | (MH "Gambia") | 639 |
| 115 | (TI Gambia OR AB Gambia) | 640 |
| 116 | (SU Gambia) | 640 |
| 117 | (MH "Guinea-Bissau") | 193 |
| 118 | (TI Guinea-Bissau OR AB Guinea-Bissau) | 241 |
| 119 | (SU Guinea-Bissau) | 193 |
| 120 | (MH "Liberia") | 576 |
| 121 | (TI Liberia OR AB Liberia) | 676 |
| 122 | (SU Liberia) | 577 |
| 123 | (MH "Madagascar") | 396 |
| 124 | (TI Madagascar OR AB Madagascar) | 550 |
| 125 | (SU Madagascar) | 396 |
| 126 | (MH "Malawi") | 3,113 |
| 127 | (TI Malawi OR AB Malawi) | 3,548 |
| 128 | (SU Malawi) | 3,113 |
| 129 | (MH "Mali") | 625 |
| 130 | (TI Mali OR AB Mali) | 867 |
| 131 | (SU Mali) | 626 |
| 132 | (MH "Mozambique") | 1,024 |
| 133 | (TI Mozambique OR AB Mozambique) | 1,285 |
| 134 | (SU Mozambique) | 1,027 |
| 135 | (MH "Niger") | 357 |
| 136 | (TI Niger OR AB Niger) | 1,004 |
| 137 | (SU Niger) | 357 |
| 138 | (MH "Rwanda") | 1,335 |
| 139 | (TI Rwanda OR AB Rwanda) | 1,585 |
| 140 | (SU Rwanda) | 1,337 |
| 141 | (MH "Sierra Leone") | 872 |
| 142 | (TI "Sierra Leone" OR AB "Sierra Leone") | 1,118 |
| 143 | (SU Sierra Leone) | 875 |
| 144 | (MH "Somalia") | 1,024 |
| 145 | (TI Somalia OR AB Somalia) | 605 |
| 146 | (SU Somalia) | 1,025 |
| 147 | (TI "South Sudan" OR AB "South Sudan") | 319 |
| 148 | (MH "Sudan") | 1,541 |
| 149 | (TI Sudan OR AB Sudan) | 1,652 |
| 150 | (SU Sudan) | 1,544 |
| 151 | (MH "Syria") | 1,549 |
| 152 | (TI Syria OR AB Syria) | 958 |
| 153 | (SU Syria) | 1,551 |
| 154 | (MH "Togo") | 210 |
| 155 | (TI Togo OR AB Togo) | 309 |
| 156 | (SU Togo) | 210 |
| 157 | (MH "Uganda") | 6,404 |
| 158 | (TI Uganda OR AB Uganda OR) | 7,155 |
| 159 | (SU Uganda) | 6,414 |
| 160 | (MH "Yemen") | 584 |
| 161 | (TI Yemen OR AB Yemen) | 651 |
| 162 | (SU Yemen) | 588 |
| 163 | (MH "Developing Countries") | 19,903 |
| 164 | (TI "developing countr*" OR AB "developing countr*") | 17,855 |
| 165 | (TI LIC# OR AB LIC#) | 1,466 |
| 166 | (SU Developing Countries) | 19,913 |
| 167 | (S85 OR S86 OR S87 OR S88 OR S89 OR S90 OR S91 OR S92 OR S93 OR S94 OR S95 OR S96 OR S97 OR S98 OR S99 OR S100 OR S101 OR S102 OR S103 OR S104 OR S105 OR S106 OR S107 OR S108 OR S109 OR S110 OR S111 OR S112 OR S113 OR S114 OR S115 OR S116 OR S117 OR S118 OR S119 OR S120 OR S121 OR S122 OR S123 OR S124 OR S125 OR S126 OR S127 OR S128 OR S129 OR S130 OR S131 OR S132 OR S133 OR S134 OR S135 OR S136 OR S137 OR S138 OR S139 OR S140 OR S141 OR S142 OR S143 OR S144 OR S145 OR S146 OR S147 OR S148 OR S149 OR S150 OR S151 OR S152 OR S153 OR S154 OR S155 OR S156 OR S157 OR S158 OR S159 OR S160 OR S161 OR S162 OR S163 OR S164 OR S165 OR S166) |  |
| 168 | S17 AND S79 AND S88 AND S175 | 913 |
| 169 | Limiters - Publication Date: 20150101-20251231 | 499 |

**Cochrane Library**

| Search | Filter | Hits |
| --- | --- | --- |
|  | Browse by Topic |  |
|  | Neonatal Care |  |
|  | Custom Range 01.01.2015 – 31.12.2025 | 265 |

**A2 Gray literature search in web search engine**

**Google Scholar**

filter: timeline 2015-2025, sort by relevance, any languages, all types of publications, patents and quotations were not included

| Search | Keywords | Hits |
| --- | --- | --- |
| 1 | health interventions to reduce neonatal mortality in Afghanistan | 10 |
| 2 | health interventions to reduce neonatal mortality in Burkina Faso | 10 |
| 3 | health interventions to reduce neonatal mortality in Burundi | 10 |
| 4 | health interventions to reduce neonatal mortality in Central African Republic | 10 |
| 5 | health interventions to reduce neonatal mortality in Chad | 10 |
| 6 | health interventions to reduce neonatal mortality in Congo | 10 |
| 7 | health interventions to reduce neonatal mortality in Eritrea | 10 |
| 8 | health interventions to reduce neonatal mortality in Ethiopia | 10 |
| 9 | health interventions to reduce neonatal mortality in Gambia | 10 |
| 10 | health interventions to reduce neonatal mortality in Guinea-Bissau | 10 |
| 11 | health interventions to reduce neonatal mortality in North Korea | 10 |
| 12 | health interventions to reduce neonatal mortality in Liberia | 10 |
| 13 | health interventions to reduce neonatal mortality in Madagascar | 10 |
| 14 | health interventions to reduce neonatal mortality in Malawi | 10 |
| 15 | health interventions to reduce neonatal mortality in Mali | 10 |
| 16 | health interventions to reduce neonatal mortality in Mozambique | 10 |
| 17 | health interventions to reduce neonatal mortality in Niger | 10 |
| 18 | health interventions to reduce neonatal mortality in Rwanda | 10 |
| 19 | health interventions to reduce neonatal mortality in Sierra Leone | 10 |
| 20 | health interventions to reduce neonatal mortality in Somalia | 10 |
| 21 | health interventions to reduce neonatal mortality in South Sudan | 10 |
| 22 | health interventions to reduce neonatal mortality in Sudan | 10 |
| 23 | health interventions to reduce neonatal mortality in Syrian | 10 |
| 24 | health interventions to reduce neonatal mortality in Togo | 10 |
| 25 | health interventions to reduce neonatal mortality in Uganda | 10 |
| 26 | health interventions to reduce neonatal mortality in Yemen | 10 |
| 27 | health interventions to reduce neonatal mortality in low-income countries | 10 |
|  |  | 270 |

**A3 List of excluded studies**

| No. | Study | Reason for exclusion |
| --- | --- | --- |
| 1 | Alene AG, Olayemi OO, Oladokun A, Berhane Y. The effect of maternity waiting homes on pregnancy outcomes in West Gojjam, northwest Ethiopia. African Journal of Midwifery & Women's Health. 2022; 16:1-13. https://doi.org/10.12968/ajmw.2022.0005 | Link to sepsis, birth asphyxia or prematurity missing |
| 2 | Ameh CA, Kerr R, Madaj B, Mdegela M, Kana T, Jones S, et al. Knowledge and Skills of Healthcare Providers in Sub-Saharan Africa and Asia before and after Competency-Based Training in Emergency Obstetric and Early Newborn Care. PLoS ONE [Electronic Resource]. 2016; 11:e0167270. https://doi.org/10.1371/journal.pone.0167270 | Implementation of intervention before 2015 |
| 3 | Ameh CA, van den Broek N. Making It Happen: Training health-care providers in emergency obstetric and newborn care. Best Practice & Research: Clinical Obstetrics & Gynaecology. 2015; 29:1077-1091. https://doi.org/10.1016/j.bpobgyn.2015.03.019 | Implementation of intervention before 2015 |
| 4 | Amsalu R, Schulte-Hillen C, Garcia DM, Lafferty N, Morris CN, Gee S, et al. Lessons learned from helping babies survive in humanitarian settings. Pediatrics. 2020; 146. https://doi.org/10.1542/peds.2020-016915L | Outcome neonatal mortality not reported |
| 5 | Andrianaivo N, Rakotovao JP, Razafimandimby E. Changing the trend of maternal and newborn mortality by basic gestures in Madagascar. Transactions of the Royal Society of Tropical Medicine and Hygiene. 2019; 113: 235-236. https://doi.org/10.1093/trstmh/trz090 | Abstract of poster presentation |
| 6 | Arabi AME, Ibrahim SA, Manar AR, Abdalla MS, Ahmed SE, Dempsey EP, et al. Perinatal outcomes following Helping Babies Breathe training and regular peer-peer skills practice among village midwives in Sudan. Archives of Disease in Childhood. 2018; 103:24-27. https://doi.org/10.1136/archdischild-2017-312809 | Implementation of intervention before 2015 |
| 7 | Armstrong C, Yui Y, Nakibuuka-Kirabira V, Bainghana P, Kyambadde R, Onwona-Agyeman K, et al. Designated neonatal resuscitation and transport team implementation improves neonatal outcomes in low resource settings. Pediatrics. 2021; 147(3):691-692. https://doi.org/10.1542/peds.147.3MA7.691 | Outcome neonatal mortality not reported |
| 8 | Atefi Y, Chen S, Farrell A, Mahmoud E. Impact of helping babies breathe training on the change in knowledge, attitude and practice among community health care workers in Jimma region, Ethiopia. Annals of Global Health. 2016; 82(3):319. https://doi.org/10.1016/j.aogh.2016.04.581 | Start of intervention not reported |
| 9 | Beressa G, Whiting SJ, Belachew T. Effect of nutrition education integrating the health belief model and theory of planned behavior on dietary diversity of pregnant women in Southeast Ethiopia: a cluster randomized controlled trial. Nutrition Journal. 2024; 23:3. https://doi.org/10.1186/s12937-023-00907-z | Outcome neonatal mortality not reported |
| 10 | Blair A, Mwanza O, Rouse M, Magid S, Simwinga L, Phiri M, et al. Evaluating a clinical mentorship intervention on maternal and neonatal complications in primary health facilities in Blantyre district, Malawi: A longitudinal analysis of the Global Action in Nursing program. International Journal of Nursing Studies. 2024; 158. https://doi.org/10.1016/j.ijnurstu.2024.104847 | Outcome neonatal mortality not reported |
| 11 | Burgoine K, Ikiror J, Akol S, Kakai M, Talyewoya S, Sande A, et al. Staged implementation of a two-tiered hospital-based neonatal care package in a resource-limited setting in Eastern Uganda. BMJ Global Health. 2018; 3:e000586. https://doi.org/10.1136/bmjgh-2017-000586 | Implementation of intervention before 2015 |
| 12 | Chan NH, Merali HS, Mistry N, Kealey R, Campbell DM, Morris SK, et al. Utilization of a novel mobile application, "HBB Prompt", to reduce Helping Babies Breathe skills decay. PLOS Global Public Health. 2023; 3:e0000705. https://doi.org/10.1371/journal.pgph.0000705 | Outcome neonatal mortality not reported |
| 13 | Chang MP, Walters CB, Tsai C, Aksamit D, Kateh F, Sampson J. Evaluation of a Neonatal Resuscitation Curriculum in Liberia. Children (Basel). 2019; 6. https://doi.org/10.3390/children6040056 | Outcome neonatal mortality not reported |
| 14 | Chimbe Phiri E, Chaputula B, Shawa E, Chiaravalli J, Sigalet E, Gabriel J, et al. A Simulation Scenario Focused on Resuscitation of a Young Infant (Neonate) for Nurses and Midwives in Malawi, a Limited Resource Country. Cureus. 2018; 10:e2673. https://doi.org/10.7759/cureus.2673 | Outcome neonatal mortality not reported |
| 15 | Dastur Mackenzie FM, Shepherd K, Rukabyarwema JP, Lissauer T. Neonatal hypothermia-still a serious problem in a low-income country. Archives of Disease in Childhood. 2019; 104(Supplement 2):107-108. https://doi.org/10.1136/archdischild-2019-rcpch.257 | Start of intervention not reported |
| 16 | de Kok B, Toe LC, Hanley-Cook G, Argaw A, Ouédraogo M, Compaoré A, et al. Prenatal fortified balanced energy-protein supplementation and birth outcomes in rural Burkina Faso: A randomized controlled efficacy trial. PLoS Medicine. 2022; 19:1-20. https://doi.org/10.1371/journal.pmed.1004002 | Outcome neonatal mortality not reported |
| 17 | Harris C, Patel H, Corn O, Harris U, Munyagwa M, Greenough A. Neonatal mortality in western uganda. Archives of Disease in Childhood. 2018; 103(Supplement 1):112-113. https://doi.org/10.1136/archdischild-2018-rcpch.268 | Start of intervention not reported |
| 18 | Heard Stittum AJ, Edwards EM, Abayneh M, Gebremedhin AD, Horn D, Berkelhamer SK, et al. Impact of an Educational Clinical Video Combined with Standard Helping Babies Breathe Training on Acquisition and Retention of Knowledge and Skills among Ethiopian Midwives. Children. 2023; 10(11). https://doi.org/10.3390/children10111782 | Outcome neonatal mortality not reported |
| 19 | Hendrixson DT, Lasowski PN, Koroma AS, Manary MJ. Newborn Mid-Upper Arm Circumference Identifies Low-Birth Weight and Vulnerable Infants: A Secondary Analysis. Current Developments in Nutrition. 2022; 6:nzac138. https://doi.org/10.1093/cdn/nzac138 | Link to sepsis, birth asphyxia or prematurity missing |
| 20 | Hulsbergen M, Abera B, Adefris M, Kassahun D, Meulenbeld M, van Nievelt S, et al. Evaluation of the Emergency Obstetric and Newborn Care training in Gondar, Ethiopia; a mixed methods study. PLOS Global Public Health. 2023; 3:e0000889. https://doi.org/10.1371/journal.pgph.0000889 | Outcome neonatal mortality not reported |
| 21 | Ilboudo B, Savadogo LGB, Traore I, Meda CZ, Herve H, Kinda M, et al. Effect of personalized home-based support for pregnant women on pregnancy outcomes: a cluster randomized trial. Journal of Public Health in Africa. 2022; 13(3). https://doi.org/10.4081/jphia.2022.1939 | Outcome neonatal mortality not reported |
| 22 | Kawaza K, Machen HE, Brown J, Mwanza Z, Iniguez S, Gest A, et al. Efficacy of a low-cost bubble CPAP system in treatment of respiratory distress in a neonatal ward in Malawi. Malawi Med J. 2016; 28:131-137. https://doi.org/10.1371/journal.pone.0086327 | Implementation of intervention before 2015 |
| 23 | Kirabira VN, Aminu M, Dewez JE, Byaruhanga R, Okong P, Van Den Broek N. Prospective study to explore changes in quality of care and perinatal outcomes after implementation of perinatal death audit in Uganda. BMJ Open. 2020; 10(7). https://doi.org/10.1136/bmjopen-2018-027504 | Implementation of intervention before 2015 |
| 24 | Kshirsagar V, Rithika D, Mohite R. A study to assess the impact of vitamin c and e intervention on biochemical profile of newborn baby with birth asphyxia. International Journal of Pharmaceutical Research. 2021; 13(2):1524-1529. https://doi.org/10.31838/ijpr/2021.13.02.219 | Intervention not conducted in LIC |
| 25 | Kukora SK, Jebessa S, Abayneh M, Metaferia G, Quinonez S. Neonatal resuscitation and airway simulation training for ethiopian pediatrics residents. Pediatrics Conference: National Conference on Education. 2019; 144. https://doi.org/10.1542/peds.144.2MA6.559 | Start of intervention not reported |
| 26 | Linner A, Westrup B, Rettedal S, Kawaza K, Naburi H, Newton S, et al. Immediate skin-to-skin contact for low birth weight infants is safe in terms of cardiorespiratory stability in limited-resource settings. Global Pediatrics. 2023; 3:None. https://doi.org/10.1016/j.gpeds.2022.100034 | Outcome neonatal mortality not reported |
| 27 | Lokangaka A, Bauserman M, Coppieters Y, Engmann C, Qazi S, Tshefu A, et al. Simplified antibiotic regimens for treating neonates and young infants with severe infections in the Democratic Republic of Congo: A comparative efficacy trial. Maternal Health, Neonatology and Perinatology. 2018; 4(1). https://doi.org/10.1186/s40748-018-0076-2 | Implementation of intervention before 2015 |
| 28 | Magge H, Chilengi R, Jackson EF, Wagenaar BH, Kante AM. Tackling the hard problems: implementation experience and lessons learned in newborn health from the African Health Initiative. BMC Health Services Research. 2017; 17:829. https://doi.org/10.1186/s12913-017-2659-4 | Implementation of intervention before 2015 |
| 29 | Marquez SP, Robinson I, Vibert YM. Mentoring birth attendants to improve skills with neonatal resuscitation in Gambia. Annals of Global Health. 2016; 82(3):529. https://doi.org/10.1016/j.aogh.2016.04.431 | Intervention period not reported |
| 30 | Medvedev MM, Brotherton H, Gai A, Tann C, Gale C, Waiswa P, et al. Development and validation of a simplified score to predict neonatal mortality risk among neonates weighing 2000 g or less (NMR-2000): an analysis using data from the UK and The Gambia. The Lancet Child and Adolescent Health. 2020; 4(4):299-311. https://doi.org/10.1016/S2352-4642%2820%2930021-3 | Outcome neonatal mortality not reported |
| 31 | Miller L, Wanduru P, Wangia J, Calkins K, Spindler H, Butrick E, et al. Simulation and team training to improve preterm birth knowledge, evidence-based practices, and communication skills in midwives in Kenya and Uganda: Findings from a pre- and post-intervention analysis. PLOS Global Public Health. 2023; 3:e0001695. https://doi.org/10.1371/journal.pgph.0001695 | Outcome neonatal mortality not reported |
| 32 | Mulinganya G, Balolebwami S, Zigabe S, Mongane J, Nianci I, Burume A, et al. Evaluation of a turbidimetric C-reactive protein assay to monitor early-onset neonatal sepsis in South Kivu (Democratic Republic of the Congo). Clinical Chemistry and Laboratory Medicine. 2021; 59(3):625-630. https://doi.org/10.1515/cclm-2020-0309 | Outcome neonatal mortality not reported |
| 33 | Namazzi G, Achola KA, Jenny A, Santos N, Butrick E, Otieno P, et al. Implementing an intrapartum package of interventions to improve quality of care to reduce the burden of preterm birth in Kenya and Uganda. Implementation Science Communications. 2021; 2:10. https://doi.org/10.1186/s43058-021-00109-w | Outcome neonatal mortality not reported |
| 34 | Ndelema B, Van den Bergh R, Manzi M, van den Boogaard W, Kosgei RJ, Zuniga I, et al. Low-tech, high impact: care for premature neonates in a district hospital in Burundi. A way forward to decrease neonatal mortality. BMC Res Notes. 2016; 9:28. https://doi.org/10.1186/s13104-015-1666-y | Implementation of intervention before 2015 |
| 35 | Niermeyer S. From the Neonatal Resuscitation Program to Helping Babies Breathe: Global impact of educational programs in neonatal resuscitation. Seminars In Fetal & Neonatal Medicine. 2015; 20:300-308. https://doi.org/10.1016/j.siny.2015.06.005 | Implementation of intervention before 2015 |
| 36 | Ntigurirwa P, Mellor K, Langer D, Evans M, Robertson E, Tuyisenge L, et al. A health partnership to reduce neonatal mortality in four hospitals in Rwanda. Global Health. 2017; 13:28. https://doi.org/10.1186/s12992-017-0252-6 | Implementation of intervention before 2015 |
| 37 | Ntwari C, Sloman R, Ntwari A, Birahinduka D. Knowledge and Skills Retention-Reducing Neonatal Mortality in Low Income Countries. Archives of Disease in Childhood. 2022; 107(Supplement 2):329. https://doi.org/10.1136/archdischild-2022-rcpch.532 | Start of intervention not reported |
| 38 | Nyishime M, Biziyaremye F, Nahimana E, Nshimyiryo A, Byiringiro S, Manirakiza ML, et al. Scaling-up the All Babies Count programme to eliminate preventable neonatal deaths in Rwanda: experiences midway through implementation. The Lancet Global Health. 2019; 7(Supplement 1):51. https://doi.org/10.1016/S2214-109X%2819%2930136-6. | Outcome neonatal mortality not reported |
| 39 | O'Connor S, Hamid A, Elsaid H, Ahmed S, Malik Y, McCarthy E, et al. Helping babies breathe in Sudan. World of Irish Nursing & Midwifery. 2016; 24:55-56. | Implementation of intervention before 2015 |
| 40 | Otolorin E, Gomez P, Currie S, Thapa K, Dao B. Essential basic and emergency obstetric and newborn care: from education and training to service delivery and quality of care. International Journal of Gynaecology & Obstetrics. 2015; 130(Supplement 2):46-53. https://doi.org/10.1016/j.ijgo.2015.03.007 | Implementation of intervention before 2015 |
| 41 | Ouedraogo P, Monfredini C, Zagre N, Papotti M, Plicco C, Tubaldi L, et al. How to progress from neonatal essential care in low income countries? The nest (neonatal essential survival tecnology) project in the Saint Camille Hospital of Ouagadougou (HOSCO). Journal of Maternal-Fetal and Neonatal Medicine. 2021; 34(Supplement 1):121. https://doi.org/10.1080/14767058.2021.1962370 | Intervention period not reported |
| 42 | Reynolds A, Zaky A, Moreira-Barros J, Bernardes J. Building a Maternal and Newborn Care Training Programme for Health-Care Professionals in Guinea-Bissau. Acta Med Port. 2017; 30:734-741. https://doi.org/10.20344/amp.8453 | Implementation of intervention before 2015 |
| 43 | Riley C, Spies LA, Prater L, Garner SL, Samra HA. Improving Neonatal Outcomes Through Global Professional Development. Advances in Neonatal Care (Lippincott Williams & Wilkins). 2019; 19:56-64. https://doi.org/10.1097/ANC.0000000000000550 | Outcome neonatal mortality not reported |
| 44 | Samuel H, Chaiken SR, Doshi U, Arora M, Caughey AB. Immediate kangaroo mother care for low birth weight babies reduces neonatal mortality: A cost-effectiveness analysis. American Journal of Obstetrics and Gynecology. 2023; 228(Supplement 1):140-141. https://doi.org/10.1016/j.ajog.2022.11.279 | Start of intervention not reported |
| 45 | Schaltz-Buchholzer F, Biering-Sorensen S, Lund N, Monteiro I, Umbasse P, Fisker AB, et al. Early BCG Vaccination, Hospitalizations, and Hospital Deaths: Analysis of a Secondary Outcome in 3 Randomized Trials from Guinea-Bissau. Journal of Infectious Diseases. 2019; 219(4):624-632. https://doi.org/10.1093/infdis/jiy544 | Implementation of intervention before 2015 |
| 46 | Schaltz-Buchholzer F, Nielsen S, Sorensen MK, Stjernholm EB, Fabricius RA, Umbasse P, et al. Effects of Neonatal BCG-Japan Versus BCG-Russia Vaccination on Overall Mortality and Morbidity: Randomized Controlled Trial From Guinea-Bissau (BCGSTRAIN II). Open Forum Infectious Diseases. 2024; 11(3). https://doi.org/10.1093/ofid/ofae057 | Link to sepsis, birth asphyxia or prematurity missing |
| 47 | Stubbe L, Philippeit A, Philippeit J, Kaukemuller L, Kruppa M, Sunder-Plassmann M, et al. Web-Based Development of Standard Operating Procedures and Midwifery Trainings at Ugandan Birth Clinic in the Framework of Implementing a Quality Improvement System for the MEWU-Midwife Exchange with Uganda. Womens Health Reports. 2024; 5:775-784. https://doi.org/10.1089/whr.2024.0068 | Link to sepsis, birth asphyxia or prematurity missing |
| 48 | Tiruneh GT, Nigatu TG, Magge H, Hirschhorn LR. Using the Implementation Research Logic Model to design and implement community-based management of possible serious bacterial infection during COVID-19 pandemic in Ethiopia. BMC health services research. 2022; 22(1):1515. https://doi.org/10.1186/s12913-022-08945-9 | Outcome neonatal mortality not reported |
| 49 | Tiruneh GT, Odwe G, K'Oduol K, Gwaro H, Fesseha N, Moraa Z, et al. Improving possible serious bacterial infection (PSBI) management in young infants when referral is not feasible: lessons from embedded implementation research in Ethiopia and Kenya. BMC Pediatrics. 2024; 24(1). https://doi.org/10.1186/s12887-024-05070-w | Outcome neonatal mortality not reported |
| 50 | Tiruneh GT, Odwe G, Kamberos AH, K'Oduol K, Fesseha N, Moraa Z, et al. Optimizing integration of community-based management of possible serious bacterial infection (PSBI) in young infants into primary healthcare systems in Ethiopia and Kenya: successes and challenges. BMC health services research. 2024; 24(1):280. https://doi.org/10.1186/s12913-024-10679-9 | Outcome neonatal mortality not reported |
| 51 | Travers CP, Carlo WA. How to Save 1 Million Lives in a Year in Low- and Middle-Income Countries. Neonatology. 2017; 111:431-436. https://doi.org/10.1159/000460512 | Implementation of intervention before 2015 |
| 52 | Tumolo M, Ruffini C, Scelsi S, Arlotta G, Boccuzzi A, Castigliani L, et al. Paediatric and neonatal training in low-resource countries: Italian Resuscitation Council and Emergency NGO together for Afghanistan. Resuscitation. 2023; 192(Supplement 1):158. https://doi.org/10.1016/S0300-9572%2823%2900709-8 | Intervention period not reported |
| 53 | Waiswa P, Pariyo G, Kallander K, Akuze J, Namazzi G, Ekirapa-Kiracho E, et al. Effect of the Uganda Newborn Study on care-seeking and care practices: a cluster-randomised controlled trial. Glob Health Action. 2015; 8:24584. https://doi.org/10.3402/gha.v8.24584 | Implementation of intervention before 2015 |
| 54 | Weinberg S, Jones D, Worku B, Kumera M, Bose C, Patterson J. Helping Babies Survive Training Programs: Evaluating a Teaching Cascade in Ethiopia. Ethiopian journal of health sciences. 2019; 29(6):669-676. https://doi.org/10.4314/ejhs.v29i6.3 | Outcome neonatal mortality not reported |

**A4 Preferred Reporting Items for Systematic reviews and Meta-Analyses extension for Scoping Reviews (PRISMA-ScR) Checklist^1^**

| **SECTION** | **ITEM** | **PRISMA-ScR CHECKLIST ITEM** | **REPORTED ON PAGE #** |
| --- | --- | --- | --- |
| **TITLE** | | | |
| Title | 1 | Identify the report as a scoping review. | i |
| **ABSTRACT** | | | |
| Structured summary | 2 | Provide a structured summary that includes (as applicable): background, objectives, eligibility criteria, sources of evidence, charting methods, results, and conclusions that relate to the review questions and objectives. | xi  Details on the background and eligibility criteria have been omitted due to limited word count and formatting requirements of the journal. |
| **INTRODUCTION** | | | |
| Rationale | 3 | Describe the rationale for the review in the context of what is already known. Explain why the review questions/objectives lend themselves to a scoping review approach. | 1-2 |
| Objectives | 4 | Provide an explicit statement of the questions and objectives being addressed with reference to their key elements (e.g., population or participants, concepts, and context) or other relevant key elements used to conceptualize the review questions and/or objectives. | 2 |
| **METHODS** | | | |
| Protocol and registration | 5 | Indicate whether a review protocol exists; state if and where it can be accessed (e.g., a Web address); and if available, provide registration information, including the registration number. | 3  Open Science Framework (OSF)  Registration number: OSF-ID [cdpr2](https://osf.io/cdpr2)  Doi: 10.17605/OSF.IO/CDPR2 |
| Eligibility criteria | 6 | Specify characteristics of the sources of evidence used as eligibility criteria (e.g., years considered, language, and publication status), and provide a rationale. | 3 |
| Information sources* | 7 | Describe all information sources in the search (e.g., databases with dates of coverage and contact with authors to identify additional sources), as well as the date the most recent search was executed. | 3-4 |
| Search | 8 | Present the full electronic search strategy for at least 1 database, including any limits used, such that it could be repeated. | 30-33, 37-46 |
| Selection of sources of evidence† | 9 | State the process for selecting sources of evidence (i.e., screening and eligibility) included in the scoping review. | 4 |
| Data charting process‡ | 10 | Describe the methods of charting data from the included sources of evidence (e.g., calibrated forms or forms that have been tested by the team before their use, and whether data charting was done independently or in duplicate) and any processes for obtaining and confirming data from investigators. | 4-5 |
| Data items | 11 | List and define all variables for which data were sought and any assumptions and simplifications made. | 3 |
| Critical appraisal of individual sources of evidence§ | 12 | If done, provide a rationale for conducting a critical appraisal of included sources of evidence; describe the methods used and how this information was used in any data synthesis (if appropriate). | n/a  In accordance with current scoping review guidelines, no critical appraisal of study quality or risk of bias was performed. |
| Synthesis of results | 13 | Describe the methods of handling and summarizing the data that were charted. | 4-5 |
| **RESULTS** | | | |
| Selection of sources of evidence | 14 | Give numbers of sources of evidence screened, assessed for eligibility, and included in the review, with reasons for exclusions at each stage, ideally using a flow diagram. | 6 |
| Characteristics of sources of evidence | 15 | For each source of evidence, present characteristics for which data were charted and provide the citations. | 7-8 |
| Critical appraisal within sources of evidence | 16 | If done, present data on critical appraisal of included sources of evidence (see item 12). | n/a  In accordance with current scoping review guidelines, no critical appraisal of study quality or risk of bias was performed. |
| Results of individual sources of evidence | 17 | For each included source of evidence, present the relevant data that were charted that relate to the review questions and objectives. | 7-13 |
| Synthesis of results | 18 | Summarize and/or present the charting results as they relate to the review questions and objectives. | 7-13 |
| **DISCUSSION** | | | |
| Summary of evidence | 19 | Summarize the main results (including an overview of concepts, themes, and types of evidence available), link to the review questions and objectives, and consider the relevance to key groups. | 14 |
| Limitations | 20 | Discuss the limitations of the scoping review process. | 17 |
| Conclusions | 21 | Provide a general interpretation of the results with respect to the review questions and objectives, as well as potential implications and/or next steps. | 17 |
| **FUNDING** | | | |
| Funding | 22 | Describe sources of funding for the included sources of evidence, as well as sources of funding for the scoping review. Describe the role of the funders of the scoping review. | 18 |

JBI = Joanna Briggs Institute; PRISMA-ScR = Preferred Reporting Items for Systematic reviews and Meta-Analyses extension for Scoping Reviews.

* Where *sources of evidence* (see second footnote) are compiled from, such as bibliographic databases, social media platforms, and Web sites.

† A more inclusive/heterogeneous term used to account for the different types of evidence or data sources (e.g., quantitative and/or qualitative research, expert opinion, and policy documents) that may be eligible in a scoping review as opposed to only studies. This is not to be confused with *information sources* (see first footnote).

‡ The frameworks by Arksey and O’Malley (6) and Levac and colleagues (7) and the JBI guidance (4, 5) refer to the process of data extraction in a scoping review as data charting*.*

§ The process of systematically examining research evidence to assess its validity, results, and relevance before using it to inform a decision. This term is used for items 12 and 19 instead of "risk of bias" (which is more applicable to systematic reviews of interventions) to include and acknowledge the various sources of evidence that may be used in a scoping review (e.g., quantitative and/or qualitative research, expert opinion, and policy document).

**References**

1.	Tricco AC, Lillie E, Zarin W, et al. PRISMA Extension for Scoping Reviews (PRISMA-ScR): Checklist and Explanation. *Ann Intern Med*. Oct 2 2018;169(7):467-473. <https://doi.org/10.7326/M18-0850>
